# Revisiting photodynamic therapy dosimetry: reductionist & surrogate approaches to facilitate clinical success

**DOI:** 10.1088/0031-9155/61/7/R57

**Published:** 2016-03-10

**Authors:** Brian W Pogue, Jonathan T Elliott, Stephen C Kanick, Scott C Davis, Kimberley S Samkoe, Edward V Maytin, Stephen P Pereira, Tayyaba Hasan

**Affiliations:** 1 Thayer School of Engineering, Dartmouth College, Hanover, NH 03755, USA; 2 Department of Surgery, Geisel School of Medicine at Dartmouth, Hanover, NH 03755, USA; 3 Department of Biomedical Engineering, Learner Research Institute, Cleveland Clinic, Cleveland, OH 44195, USA; 4 Institute for Liver and Digestive Health, University College London, London, NW3 2QG, UK; 5 Wellman Center for Photomedicine, Massachusetts General Hospital, Harvard Medical School, Boston, MA 02114, USA

## Abstract

Photodynamic therapy (PDT) can be a highly complex treatment, with many parameters influencing treatment efficacy. The extent to which dosimetry is used to monitor and standardize treatment delivery varies widely, ranging from measurement of a single surrogate marker to comprehensive approaches that aim to measure or estimate as many relevant parameters as possible. Today, most clinical PDT treatments are still administered with little more than application of a prescribed drug dose and timed light delivery, and thus the role of patient-specific dosimetry has not reached widespread clinical adoption. This disconnect is at least partly due to the inherent conflict between the need to measure and understand multiple parameters *in vivo* in order to optimize treatment, and the need for expedience in the clinic and in the regulatory and commercialization process. Thus, a methodical approach to selecting primary dosimetry metrics is required at each stage of translation of a treatment procedure, moving from complex measurements to understand PDT mechanisms in pre-clinical and early phase I trials, towards the identification and application of essential dose-limiting and/or surrogate measurements in phase II/III trials. If successful, identifying the essential and/or reliable surrogate dosimetry measurements should help facilitate increased adoption of clinical PDT. In this paper, examples of essential dosimetry points and surrogate dosimetry tools that may be implemented in phase II/III trials are discussed. For example, the treatment efficacy as limited by light penetration in interstitial PDT may be predicted by the amount of contrast uptake in CT, and so this could be utilized as a surrogate dosimetry measurement to prescribe light doses based upon pre-treatment contrast. Success of clinical ALA-based skin lesion treatment is predicted almost uniquely by the explicit or implicit measurements of photosensitizer and photobleaching, yet the individualization of treatment based upon each patients measured bleaching needs to be attempted. In the case of ALA, lack of PpIX is more likely an indicator that alternative PpIX production methods must be implemented. Parsimonious dosimetry, using surrogate measurements that are clinically acceptable, might strategically help to advance PDT in a medical world that is increasingly cost and time sensitive. Careful attention to methodologies that can identify and advance the most critical dosimetric measurements, either direct or surrogate, are needed to ensure successful incorporation of PDT into niche clinical procedures.

## 1. Introduction

In this review, an analysis was undertaken about how dosimetry issues may improve or may inhibit the success of photodynamic therapy (PDT) in clinical procedure development, and how strategically reducing the overhead of dosimetry can benefit trials as they progress beyond the safety assessment stage. PDT is a light activated chemotherapy (Kessel 1992, Dougherty *et al* 1998, Celli *et al* 2010, Agostinis *et al* 2011), used to kill tissue, in which optically activated photosensitizer molecules are excited to a triplet state, which is quenched by molecular oxygen, producing singlet oxygen. Singlet oxygen can be produced in high yield by the cycling of the photosensitizer molecules from significant amounts of light irradiation, and ultimately tissue destruction occurs. The light delivery is usually highly confined to the prescribed treatment volume of tumor tissue, and the factors that affect outcome are the amount of each key component throughout the volume to be treated, including the photosensitizer, light fluence delivered (Wilson and Patterson 1986, 2008) and availability of oxygen for quenching (Foster and Gao 1992, Zilberstein *et al* 1997, Georgakoudi and Foster 1998b, Edrei and Kimel 1999, Bajgar *et al* 2014). There are many different photosensitizers (Ochsner 1997), each of which has particular strengths and weaknesses, and has undergone niche application developments in treatment (Hammer-Wilson *et al* 1999). While there are very successful clinical treatments today (Patel *et al* 2014), comparatively these are few relative to the vast body of published clinical trials where PDT has been evaluated.

Unfortunately, the most common outcome in PDT clinical trials has been the failure of each procedure to reach a critical mass of adoption by practitioners. In cases where advanced phase I/II clinical trials have not led to adoption, some of these failures are tied to the delivery and control processes required, as well as the variability in clinical responses due to uncontrolled dose delivery, resulting in a lack of perceived value in the technique. In commercial applications, it is generally believed that the simplest PDT procedures, delivered without the need for dosimetry measurements or tools (i.e. PDT for skin actinic keratosis) are the preferred way to achieve clinical acceptance. This is a logical perspective; however, delivery problems or unpredicted results with these spartan procedures often go unexplained. Ultimately, this can lead to the lack of wider adoption in that clinical specialty area, because the treatment is associated with unpredictable response rates or a failure to control the disease. Dosimetry describes the quantitative planning and verification processes used to prescribe and verify a patient treatment, which will ensure that the targeted areas are given appropriate dose to kill the tissue, and to prevent over-treatment to normal tissues, avoiding morbidity issues (e.g. stricture formation from esophageal PDT) (Wilson *et al* 1997). From this perspective, dosimetry is undoubtedly one of the most important issues for patient safety in localized physical treatments such as PDT. Both under-treatment of the targeted tissue and/or over-treatment of surrounding healthy tissue at risk can lead to lack of enthusiasm for PDT treatment adoption. In both cases, the judicious use of a clinically acceptable dosimetry process, which is not too cumbersome to implement, and ideally utilizes clinically known procedures for measurements, would provide assurance that treatment was being delivered as intended. Dosimetry research tends to push towards an explicit solution where all possible parameters are measured—yet, a heavy-handed approach can also lead to lack of clinical enthusiasm due to the complexity in implementing the additional measurements. In this review, we propose what we believe is a possible balancing point between using complete dosimetry in mechanistic studies and even early phase trials, and the minimally-sufficient approach to dosimetry in later stage clinical trials and clinical practice. This is done by determining the key factor governing dose delivery and either directly measuring that in all cases, or finding an alternative clinical surrogate measurement.

Unfortunately, because the deposition of PDT dose to biological tissue occurs at the micro-environmental level (i.e. based on the interactions of photosensitizer, molecular oxygenation, and light distribution within cellular ultrastructural components) (Evans *et al* 2011, Anbil *et al* 2013, Rizvi *et al* 2013) the variability in cumulative delivery can be high on both the microscopic scale as well as between subjects (Zhou *et al* 2006). In this review, we outline efforts to apply explicit dosimetry to map the distributions of light delivery, photosensitizer concentration, blood flow and tissue oxygen concentration. Secondarily, we briefly review efforts towards implicit dosimetry to measure or map photosensitizer fluorescence photobleaching or singlet oxygen luminescence, as direct end products that are more closely related to dose. Finally, we examine the potential to use conventional clinical modalities, such as optical imaging, contrast x-ray computed tomography (CT), ultrasound (US), magnetic resonance imaging (MRI), and tissue and blood sampling, as patient-specific surrogate PDT dosimetry tools. Because these modalities are widely used for patient workup, management of cancer, and assessment of treatment efficacy, their adaptation to PDT dosimetry could streamline the delivery and control of PDT.

Dosimetry has been demonstrated to be an essential mechanistic tool to understanding PDT therapeutic dose, and a means for planning optimal light and drug doses for specific organ-tumor-photosensitizer combinations. Yet, lessons from prior studies are not always adopted in translational research. For example, while PDT of bladder (Star *et al* 1987, Marynissen *et al* 1989, Jocham *et al* 1990) and esophageal cancer (Panjehpour *et al* 2005, Mackenzie *et al* 2008) was shown to be problematic when the light delivery device is not properly positioned in the organ, subsequent clinical trials have been conducted without careful application of this critical safety goal. Overtreatment at the wrong site of a hollow organ can lead to perforation failure; damage to the muscular-collagen support layers can lead to stricture and severe morbidity. Treatment with PDT to the pleural cavity has been demonstrated, and dosimetry is critically important to avoid over treatment to critical structures (Friedberg *et al* 2012, Zhu *et al* 2015). Additionally, over treatment is more problematic when using some of the more potent photosensitizers, such as Foscan, Photofrin or Verteporfin, as they can be present in very high concentrations at certain times after injection (Wenig *et al* 1990, Kubler *et al* 1999, Sunar 2013). Avoiding overtreatment or under treatment is a critical issue that deserves close attention when determining the appropriate dosimetric parameter that needs to be measured on each patient.

**Figure 1.**
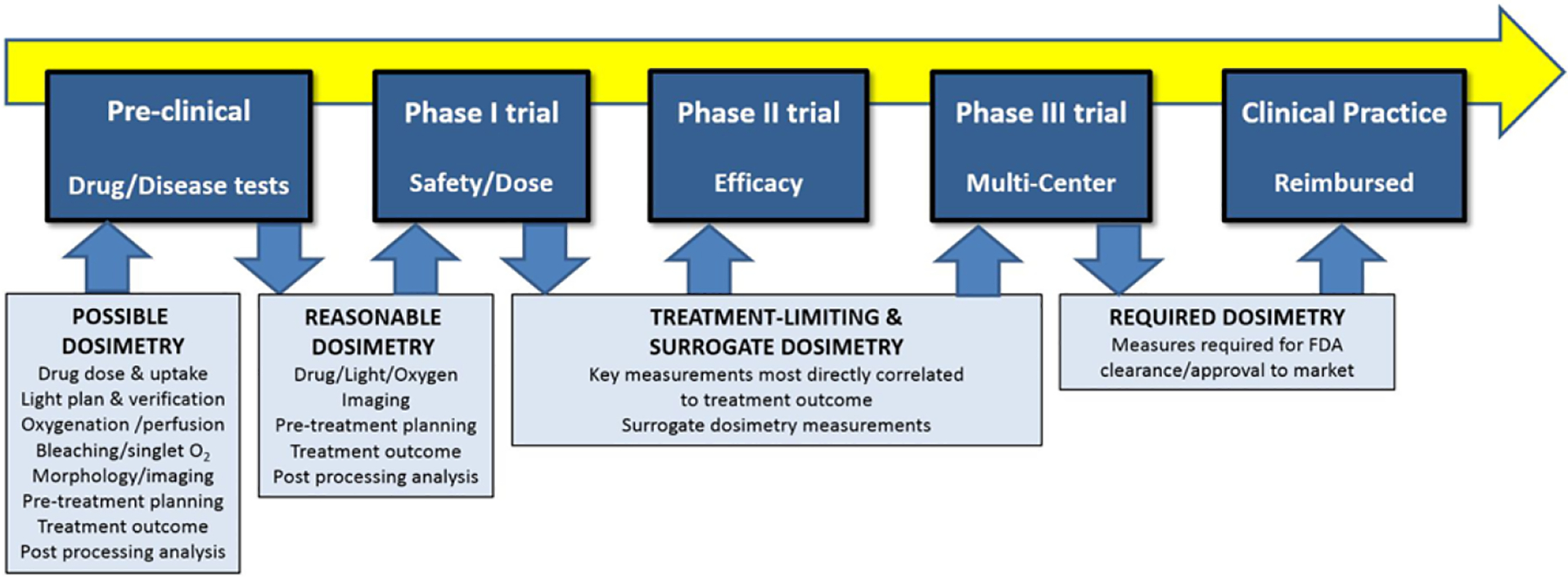
The shift from pre-clinical evaluation, through Phase I, II, and III clinical trials, and finally to accepted clinical practice is illustrated with the advocated shifts in dosimetry goals throughout the pathway. While a comprehensive dosimetry approach might be used in pre-clinical work to mechanistically inform practice, the transition to Phase I trial requires that reasonably efficient methods of dosimetry be implemented, and in the future the goal should be to shift towards treatment limiting dosimetry and correlated surrogates. Eventually, these might become the required dosimetry in approved/cleared treatments, going beyond basic prescriptions of light and drug doses.

While concerns about PDT dosimetry are well founded, there is also a concern that clinical adoption will be impeded as delivery monitoring and control becomes more cumbersome. Clinical efficiency and cost effectiveness are becoming primary factors in translating new treatments in a rapidly changing landscape of healthcare delivery. An ideal clinical development flow diagram for PDT is illustrated in figure 1. In this paradigm, a comprehensive dosimetry approach is implemented in the pre-clinical mechanistic research stage, but as development shifts to clinical trials in phases I through II and III, the goal correspondingly changes to window down the necessary-but-sufficient factors that reliably predict treatment outcome. All too often this is confused with eliminating dosimetry altogether, yet the key issue is to determine the one or two predominant factors that affect treatment outcome, and develop clinically acceptable ways to measure these. Further, in this review, we propose that focusing on readily-translatable surrogate measures of dosimetry could facilitate clinical adoption more effectively than explicit or even implicit measures in some cases. We also suggest that overt patient-specific explicit dosimetry methods might actually impede clinical adoption of PDT because of the overhead cost required by both companies and physicians. Admittedly, this argument must be taken within the context of each individual treatment and the risk/benefit ratio of the treatment. In this review, we outline areas where surrogate and treatment-determining dosimetry methods may make the most sense for future PDT trials.

## 2. PDT dosimetry approaches

### 2.1. Explicit dosimetry

Explicit dosimetry is the approach of directly measuring all pertinent parameters that contribute to the PDT treatment, or at least those that dictate the limits to treatment efficacy for the organ disease and photosensitizer used, as outlined in figure 1 (box at far bottom left). One of the key goals in explicit dosimetry is to determine those parameters that can be measured without prohibitively invasive clinical procedures. An example of this is to understand that measuring the distribution of photosensitizer and light in a solid tumor can be challenging without direct access to the tissue interior. Such direct measurements are routinely used in clinical trials of cancers with good access, such as in the skin, or for treatments where highly invasive procedures are common, such as head and neck, prostate and intra-uterine cancer. The measurement and calculation of light delivery is arguably the origin of modern approaches toward controlling PDT dose, with several groups attempting solutions in various clinical trials throughout the 1980s (Grossweiner 1986, Powers and Brown 1986, Werkhaven *et al* 1986, Wilson *et al* 1986, Grossweiner *et al* 1987, Potter *et al* 1987, Star *et al* 1987, Arnfield *et al* 1989). In this era, most of the approaches to dosimetry were focused around either direct measurement of the light fluence delivered in the tumor region or border, or computational modeling of the light fluence in the tumor, to predict the total integrated light dose within a clinically prescribed volume. In the 1990s, the approaches to dosimetry diversified considerably, as did the number of available photosensitizers, and so more focus was placed on direct measurement of the light fluence rate *in vivo* and the drug concentration where feasible (D’Hallewin *et al* 1992, Foster and Gao, 1992, Marijnissen *et al* 1993, Heier *et al* 1995, Beyer 1996, Braichotte *et al* 1996, Svaasand *et al* 1996, Tromberg *et al* 1996, Bays *et al* 1997, Wilson *et al* 1997, Lilge *et al* 1998, Tan *et al* 1999). The match between computational work and experimental measurements of light fluence was more routinely used in clinical trials. By the early 2000s, several groups had developed advanced delivery and monitoring systems that were fully integrated or were controlled by a medical physics team (Johansson *et al* 2002, van Veen *et al* 2002, Boere *et al* 2003, Radu *et al* 2003, Dickey *et al* 2004, van Veen *et al* 2006, Zhu and Finlay 2006, Johansson *et al* 2007, Swartling *et al* 2010, Zhu 2012), and pilot commercialization had emerged.

Complete dosimetry is now fairly widely recognized as a very challenging goal, with multiple probe locations required, intensive computational modeling for those areas between the control points, and geometrical problems in accurately assessing these values. Still, as a research pursuit, these developments show the major challenges of accurate dosimetry in PDT. However, we argue here that this brute force approach to dosimetry may be one of the factors leading to a lack of enthusiasm, due to the high overhead required to deliver and monitor treatment. This may be a controversial issue, but requires consideration since it is well known that PDT with explicit dosimetry has never seen clinical acceptance in a widespread, reimbursable manner. Another problem may be that a dedicated dosimetry system for PDT, developed for the single-use indication for PDT only, is inherently less attractive than a standard imaging or assay system that can be used for a broader range of procedures and is widely adopted in the clinical specialty. This is not to say that dosimetry is not needed, but rather a staged approach must be designed, which strategically finds those dosimetry parameters which dominate control of the process and could then be reduced to the minimum essential measurements or used to define surrogate measures of them.

Measurement of photosensitizer (PS) concentration in most clinical trials has been handled as a traditional drug delivery issue, whereby the prescribed injection or topical delivery dose is administered and bulk measurements of PS in serum and tissue are made. More recent efforts have focused around direct *in situ* verification of photosensitizer through online fluorescence measurement (Braichotte *et al* 1996, Farrell *et al* 1998a, Boere *et al* 2003, Yang *et al* 2003, Valentine *et al* 2013, Kanick *et al* 2014, Mallidi *et al* 2014) of each patient. This is especially important to understand and accommodate the wide variability in photosensitizer uptake/production between subjects and even within a given lesion. While early trials intentionally focused on plasma sampling and tissue extraction measurements of PS, later studies have focused on *in situ* measurements using the fluorescence properties of the PS. Several fiber probe systems exist for PS measurement in tissue, with varying levels of robustness to variations in tissue optical properties. Additionally, camera-based systems have been used largely for skin imaging (Cubeddu *et al* 2000, Fischer *et al* 2001, Ericson *et al* 2004, Tyrrell *et al* 2010a, Andrade *et al* 2014), and tomographic systems to image sub-surface PS distribution have been studied (Johansson *et al* 2007, Swartling *et al* 2010, Flynn *et al* 2013, Rollakanti *et al* 2013). The emergence of fluorescence-guided surgery has been a lateral development, significantly advancing the technologies available for imaging fluorescence in clinical settings, and this is expected to grow in the coming years.

A final obvious measurement in dosimetry is the oxygen level. However this may be the most elusive of all, because oxygen concentration varies spatially at the microscopic level, and can change dynamically throughout the treatment (Tromberg *et al* 1990, Foster and Gao, 1992, McIlroy *et al* 1998, Georgakoudi and Foster 1998b, Kelleher *et al* 2004, Zhu *et al* 2005, Woodhams *et al* 2007, Jarvi *et al* 2011, Weston and Patterson 2013). Many research efforts to measure parameters related to oxygen concentration have been reported, such as blood hemoglobin oxygen saturation (SO_2_) (Pham *et al* 2001, Solonenko *et al* 2002, Wang *et al* 2004, Thompson *et al* 2005, Wang *et al* 2005, Zhu *et al* 2005, Yu *et al* 2006, Wang *et al* 2007, Kruijt *et al* 2009), tissue partial pressure (pO_2_), and blood flow parameters. While each of these measurements is feasible in a laboratory setting, obtaining useful measurement in humans is challenging on a macroscopic scale, especially as values change dynamically during treatment. It is nearly impossible to acquire enough information that enables adequate assessment of the extreme heterogeneity present (Vaupel and Kelleher 2010). More innovative measures of oxygenation in the tissue, such as luminescence lifetime measurements of the photosensitizer (from quenching processes which are sensitive to ground state oxygen), could have value in understanding the regional variations in oxygenation; however these are currently restricted to surface imaging or diffuse imaging (Lebedev *et al* 2009, Wilson *et al* 2012).

### 2.2. Implicit dosimetry

The idea of implicit dosimetry has been a topic of advanced research for a number of years, and was eloquently summarized by Wilson *et al* (1997). The overall goal of dosimetry is to measure all relevant, individual parameters which, when added together, could provide an integrated parameter that correlates closely to treatment dose. What makes implicit dosimetry even more important is the observation that some previously non-measurable effects also seem to contribute to the dose delivery in non-linear ways. Examples of this are: (i) light dose rate & fractionation, (ii) micro-localization of the photosensitizer in the vasculature versus tumor parenchyma, (iii) cellular organelle localization effects, or (iv) whole body immune response to local PDT damage. Thus, a true appreciation of the complexity of PDT dosimetry suggests it may be impossible to measure all pertinent factors affecting dose delivery as a predictor of biological response.

The ultimate goal of PDT is the photochemical production of toxic species, and the most direct measurements of this production are thought to be of singlet oxygen generation, and biological damage occurring directly in the tissue (Douzou 1972, Niedre *et al* 2005, Jarvi *et al* 2006, 2012, Laubach *et al* 2008, Lee *et al* 2011, Mallidi *et al* 2014). For singlet oxygen, the measurement of which has been the focus of an enormous amount of engineering work, the most detectable signal is its luminescence at 1270 nm. The lifetime and amplitude of this emission are quenched by interaction with proteins, so direct measurement of the lifetime provides an estimate of the deposited PDT dose as the treatment occurs. Integrating this signal through the treatment is required to provide a metric related to dose. Also, intermittent measurement of the species can give information about oxygen limitations in treatment. While intense research is ongoing in this field, one could argue that a practical system to provide robust singlet oxygen dosimetry does not yet exist.

**Figure 2.**
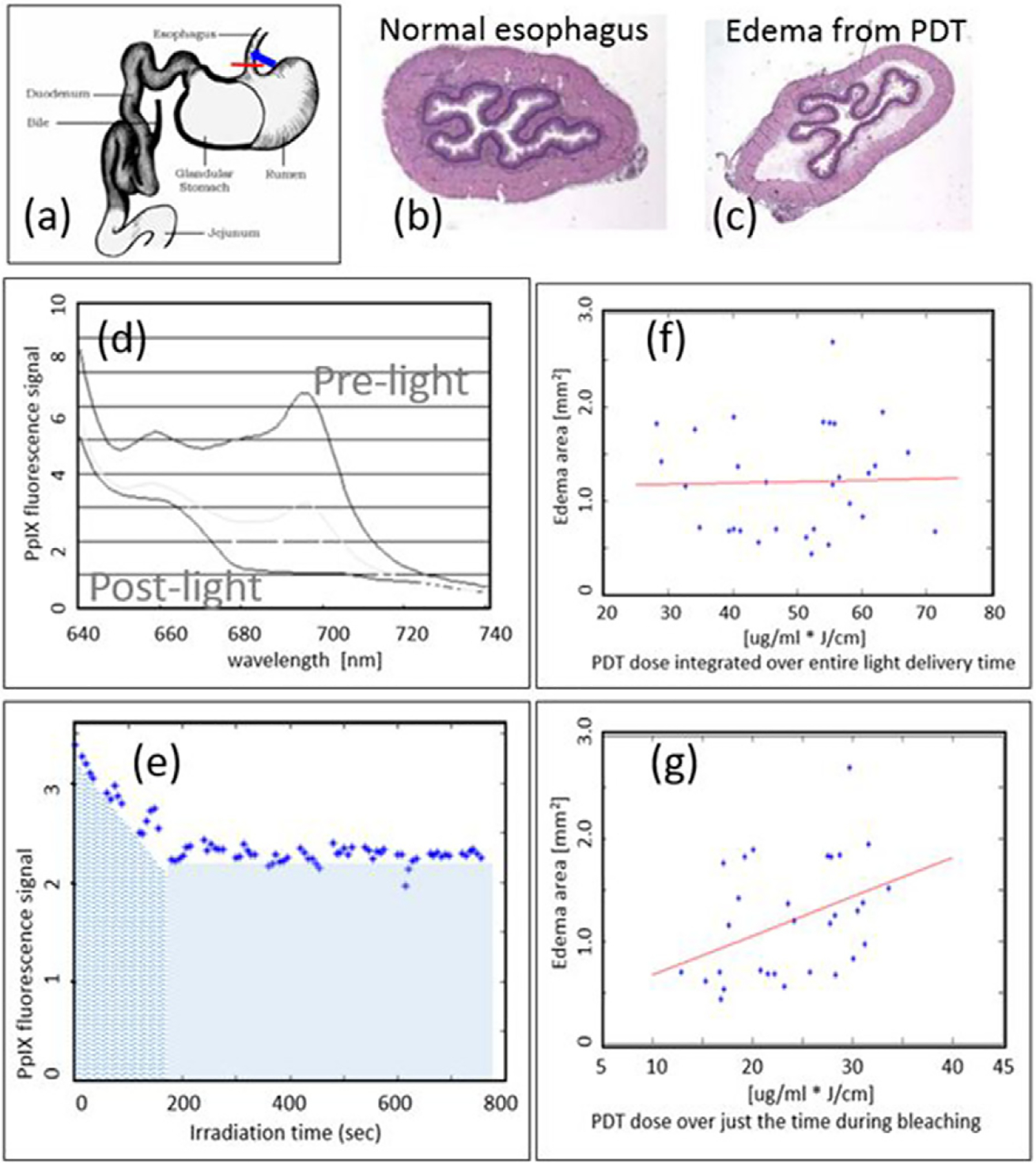
Rat esophagus was used to study PDT dosimetry and response for ALA-PpIX treatments (reproduced with permission from Sheng 2006 and Sheng *et al* 2007) (a). The histology images of each esophagus (b) were used to assay response, and the edema resulting from PDT (c). The fluorescence during treatment was spectrally resolved (d) and quantified as a function of time (e). Then the signal was tested for correlation to the edema area, and when the total fluorescence over the treatment time was used (f), there was no correlation (all blue area in (e)); yet when only the signal during the bleaching phase was used, a correlation became apparent (g) (just the blue wavy line area in (e)).

A good example of the value of implicit dosimetry comes in PpIX-based PDT, where fluorescence from PpIX can be directly measured, ideally with a spectrally resolved detection system during light irradiation. Interestingly the loss of fluorescence with PpIX is known to be quite rapid during treatment, and this photobleaching is well known to represent the time region where the maximal dose delivery occurs (Georgakoudi *et al* 1997, Farrell *et al* 1998a, Iinuma *et al* 1999, Kunz and MacRobert 2002, Zeng *et al* 2002). Studies have shown *in vivo* that the fluorescence loss integrated over the time interval of photobleaching is predictive of damage to the tissue, and further light treatment beyond this has little value (Sheng *et al* 2007), as shown in figure 2. This is a good example of how direct measurement of all parameters such as light delivery, the PpIX present, and tissue oxygenation would be not only challenging, but also insufficient to predict the treatment response. To be predictive, these parameters would have to be input into a dosimetric model which factors in photobleaching and its role as well, as shown by Wang *et al* (2010). Thus, the simpler idea of reducing all of this down to a direct measurement of integrated photobleaching signal is very attractive, assuming or verifying that this parameter directly correlates to the desired treatment outcome in a clinical trial. This has only been validated extensively for PpIX PDT.

Clinical manifestations of this implicit dosimetry approach have been demonstrated in humans through a few phase 1 clinical trials. Valentine *et al* (2011) demonstrated significant photobleaching existed in all of 40 patients who responded to ALA or methyl-AL based PDT, and later (Valentine *et al* 2013) that modeling of this could allow interpretation of the dose delivery. In the work of Ericson *et al* (2004), it was shown in 37 patients that lower fluence rates were better correlated to superior initial treatment outcome. Also the photobleaching rate was inversely related to the fluence rate, with a threshold beyond which no further bleaching occurred, validating the potential for using photobleaching as an implicit measure of *in vivo* dose in humans. Cottrell *et al* (2008), took this concept to the next level by completing a prospective study of implicit dosimetry in 26 patients, showing that the fluence required to bleach PpIX was directly related to the irradiance used, and applied the active dosimetry concept of increasing the irradiance in a separate cohort. Mallidi *et al* (2014) compared photobleaching dosimetry to direct singlet oxygen measurements before and after PDT in 26 human subjects, and demonstrated that the treatment outcome was better predicted by the photobleaching metric. It should be noted however that while this approach is important for PpIX-based PDT, this is a particular type of photosensitizer which happens to photobleach rapidly, and these relationships certainly may not hold for most other sensitizers as the photobleaching rate varies considerably between compounds. (Svanberg *et al* 1998, Fischer *et al* 2001, Ericson *et al* 2005). Research into the potential value of photobleaching itself is widespread, but despite its promise, has not seen widespread adoption at this time.

## 3. Ongoing issues in PDT dosimetry

### 3.1. Uncertainties about photosensitizer concentration, location and effect in vivo

Perhaps the best success story with PDT, at least in terms of the largest commercial success, has been the use of aminolevulinic acid (ALA) and its related hexyl and methyl esters to induce protoporphyrin IX (PpIX) (Kennedy and Pottier 1992) for the treatment of actinic keratosis (AK) and squamous cell carcinoma (SCC). There are currently several commercial suppliers of topical ALA and ALA derivatives for skin application. The solution or cream is applied to the area to be treated and after a period of hours, light irradiation is used for PDT effect. The problem with this therapy is that while approved and widely adopted, it can have extremely variable response rates (Dognitz *et al* 2008, Gerritsen *et al* 2009, Sharwani and Alharbi 2014, Lerche *et al* 2015), which is thought to be linked to large variations in PpIX production within lesions. The origins of this variation are still unclear, but may be related to ALA penetration, or simply to biological variations in the lesions (table 1).

For vascular-injected photosensitizers, there are well-documented issues around understanding and controlling the degree to which the photosensitizer leaves the vascular space and penetrates the tumor tissue (table 1). This difference in microscopic localization is very important as it has been shown that changes during treatment, or at different phases of localization, result in strong changes in biological effect (Fingar *et al* 1992a, 1992b, Fingar *et al* 1999, Chen *et al* 2003, 2005a, 2008). Understanding and controlling this variability is further confounded by the competing time-dependent processes of (i) drug extravasation into tissue, and (ii) systemic clearance through normal physiological filtering processes. Some studies have demonstrated that mTHPC plasma concentration can increase over time (Glanzmann *et al* 1998, Chen *et al* 2000, Cramers *et al* 2003, de Visscher *et al* 2011) making prediction of the *in vivo* concentration complex. Also, it has been shown that dynamic changes to the vessels can occur in the initial phases of PDT activation, and these can affect the overall dose delivery process (Chen *et al* 1998, Pogue *et al* 2001a, Chen *et al* 2003, Wang *et al* 2004, Wang *et al* 2005, Yu *et al* 2006). Finally, vascular-cellular distribution kinetics are also superimposed upon an even smaller, micro-localization processes that occur where the PS associates within the cells. Microscopic re-localization to critical membranes and organelles can also radically affect the dose sensitivity (Strauss *et al* 1995, Georgakoudi and Foster 1998a, Seidl *et al* 2001, Pogue *et al* 2001b, Krieg *et al* 2003, Ji *et al* 2006, Osaki *et al* 2006, Sasnouski *et al* 2006, Kunz *et al* 2007), so that raw measurements of bulk concentration may not be representative of the impact of dose delivered. Furthermore, the above parameters are PS-specific for each treatment situation. These complex issues are not easily solved. Therefore, while fascinating areas for research, they point to the central issue discussed, namely that absolute PDT PS dosimetry may be almost uncontrollable in many clinical settings.

### 3.2. The challenge of measuring photosensitizer, light and oxygen in bulk tissues

Solid tumor PDT is perhaps one of the most challenging for dosimetry, because of the need to measure explicit dose parameters within the lesion, while they potentially vary over time. Invasive systems exist (Zhu *et al* 2005, Du *et al* 2006) but have not achieved market success yet. The need to predict between measurement points is still a challenge, typically solved with approximation modeling. For example, optical fluence measurements can be sampled, and diffusion theory or Monte Carlo modeling can be used to predict between the measurement points (Johansson *et al* 2007, Li and Zhu 2008, Swartling *et al* 2010). Additionally, fluorescence tomography has been demonstrated to measure PS distribution in deep tissue structures (Yu *et al* 2006, Johansson *et al* 2007, Wang and Zhu 2009, Swartling *et al* 2010, Flynn *et al* 2013), yet high spatial resolution through thick tissues is perhaps an insolvable problem. Taken as a whole, explicit 3D systems for PS exist and could be implemented more widely, but their output is still limited to regional approximations, and the challenges of daily implementation would require trained technical staff to operate complex instrumentation and to interpret the data.

Oxygen is critical for successful PDT (Fingar *et al* 1987, Henderson and Fingar, 1987, Wieman and Fingar 1992), yet the search for a useful and accurate way to measure tumor oxygenation has gone on for many decades (Vaupel *et al* 2005). The most important feature of this research challenge is the fact that variations in oxygenation occur at the capillary-tissue level, and thus the oxygenation varies by orders of magnitude every few tens of microns in tissue. This fact, combined with the extreme heterogeneity of tumors, means that obtaining highly-resolved maps of oxygenation throughout an entire tumor is not feasible. That said, dozens of methods to measure oxygen or oxygen-related parameters on a larger scale have been tried. Well controlled data certainly exist to show that oxygen is required (Henderson and Fingar 1987, Fingar *et al* 1988, Henderson and Fingar 1989, Fingar *et al* 1992a, Sitnik *et al* 1998, Henderson *et al* 1999), and that oxygenation can globally change during therapy, and that enhancement of blood flow or tumor oxygenation can improve response to PDT. However, as with radiation therapy, the actual implementation of routine oxygen monitoring during therapy has remained elusive, largely because of the intractability of the problem (Pogue *et al* 2001a). Local measurements can still be done, and on average can show reasonable trends. However, the introduction of systems such as the Eppendorf electrode were potentially inhibitory to clinical acceptance, owing to the extremely invasive nature of measurement (Vaupel *et al* 2006, 2007).

Singlet oxygen luminescence dosimetry has been widely pursued as a potentially superior alternative to oxygen measurement, as it would provide a more comprehensive measure of the total dose delivered (Patterson *et al* 1990a, Niedre *et al* 2002, Jarvi *et al* 2006, Wei *et al* 2008, Jarvi *et al* 2011, Lee *et al* 2011, Mallidi *et al* 2014). Yet, as with oxygen, the singlet oxygen signal should be assumed to vary on the distance scale of 10’s of microns, and to vary dynamically throughout treatment. Significant research efforts are ongoing, and recent technological advances in mid-IR detection have made this seem more possible (Jarvi *et al* 2011, Lee *et al* 2011, Mallidi *et al* 2014). Yet, implementation of singlet oxygen detection on anything other than surface tissues seems challenging to implement in a clinically acceptable setting. Research and development efforts in this area are still progressing however, and it is quite possible that probe and surface imaging systems will advance into practical use.

### 3.3. Complete interpretive models of photodynamic dose

The goal of a physical theoretical model for dose delivery has been developed in many studies over the last several decades. Given the role of physicists in providing the basic tools and interpretations of dosimetry, the goal of providing a complete computational framework for estimating dose has been a long desired ideology, and the individual components of this model are briefly mentioned here. Early estimates of light dose spread in tissue are the first major theoretical part of this to be solved computationally with diffusion or Monte Carlo modeling (Wang *et al* 2010), but then this must be integrated with theoretical physical chemistry modeling of the active photosensitizer concentration, and the conversion of excited triplet state oxygen to singlet oxygen through collisional quenching. The added complexity of singlet oxygen then photobleaching the original photosensitizer is an important feature, which can lead to a mixture of photosensitization effects. Finally, the existence of a threshold dose in PDT effect has been validated both *in vitro* (Georgakoudi *et al* 1997, Coutier *et al* 2001) and *in vivo* (Patterson *et al* 1990b, Lilge and Wilson 1998, Farrell *et al* 1998b), demonstrating that the prediction of dose itself also must be interpreted relative to the presence of a non-linear step function before cellular destruction occurs (Wang *et al* 2010). However, there are features of photochemical oxygen depletion and biological blood flow reduction which are negative feedback aspects that can occur during the light delivery, which cannot be reliably modeled without direct measurement of these parameters, making physical modeling by itself incomplete (Foster and Gao 1992, Chen *et al* 2003).

While the tools to measure all these things are complex, one of the major goals of complete interpretive modeling would be to allow a reduced number of measurements, and yet still provide accurate dosimetry estimates. Thus, the idea of developing a complete interpretive model is not inconsistent with the idea of reducing PDT dosimetry measurements and streamlining the dosimetry process as clinical trials advance. Indeed, it will be important for interpretive modeling to be present and that surrogate or reduced dosimetry measurements agree with quantitative models in order to validate the effects seen. However, while PDT photochemical dose prediction is important, PDT response which includes all the biological effects is what actually matters for clinical trials. As such, the next section describes these issues which ultimately could have a heavy impact on the role of measurements and quantitative models, in cases where biological responses do not directly agree with quantitative photodynamic dose estimates.

### 3.4. Biological modifiers of dose response

Factors that modify tissue response to PDT have been well documented, some of which can alter the biological response to treatment, and may or may not correlate with direct physical dosimetry measurements. These types of modifisers can be divided into (i) pre-treatment modifiers which prepare the tissue for higher damage from a fixed dose, and (ii) biological factors which affect response post-treatment. The pre-treatment modifiers are factors that affect blood flow and perfusion, or factors which affect photosensitizer uptake, production or localization in the tissue to be treated.

Few clinical studies have examined vascular flow modifiers, although many pre-clinical studies have looked at this. In general, increases in tumor perfusion are thought to be good for improved delivery of PS and oxygen to the tumor. The radiobiology research world has examined this for decades without much clinical success, largely because of the fact that tumor vessels are thought to be unresponsive to modifiers, whereas normal vessels are much more responsive (Thews *et al* 2000, 2001). External modifiers such as heat, chemotherapy or radiation can induce changes in interstitial pressure, which can induce increased transvascular delivery (Bicher *et al* 1980, Lin and Song 1993, Song *et al* 1996, Vujaskovic *et al* 2000). Alternatively, mild PDT itself can induce improved transvascular delivery if done carefully, allowing for improved overall PDT delivery and response (Chen *et al* 2003, 2005a, 2005b).

Pre-treatment cellular modifiers have been examined in the recent decade, with a particular attention to PpIX production, based upon differentiating agents (Schick *et al* 1995, Ortel *et al* 2002, Ickowicz Schwartz *et al* 2004, Akilov *et al* 2008, Anand *et al* 2013). These agents are applied in the days and weeks prior to PDT (Anand *et al* 2011) and are thought to alter the transcription pathways for enzyme production to enhance the PpIX production pathway. Alternatively chemical modifiers such as iron chelators have been applied (Curnow *et al* 1998, Choudry *et al* 2003, Blake *et al* 2011), to reduce the available iron and halt the conversion of PpIX to heme, thereby yielding a larger amount of end product (PpIX). These biological modifiers produce changes in treatment response that, in addition to the alterations in PS levels (PpIX), reflect changes in other biochemical processes such as an increased activity of cell death pathways such as apoptosis (Anand *et al* 2011). While the effect of this biological modification could be measured by fluorescence and photobleaching dosimetry, their effect may not always be picked up by surrogate dosimetry metrics, and so care is required to consider this.

Finally, perhaps the largest biological effect that factors into PDT treatment is the subsequent immunological responses, and changes that occur in expression pathways of the surviving cells (De Goeij *et al* 1975, Canti *et al* 1994, Hryhorenko *et al* 1998, Wong *et al* 2003, Brackett 2011, Kammerer *et al* 2011, Shams *et al* 2015). Although these reactions have been well documented in recent decades, our understanding of these effects across a range of tumor lines is still not at a level that can be applied to clinical treatment. Despite this, there is a widespread belief that immune responses after PDT could be as important as the original PDT dose itself (Gollnick 2012, Korbelik and Hamblin 2015, Shams *et al* 2015). Developing the ability to image or assay changes in immunological and cellular signaling is a major part of PDT research today, and may well become a critical piece of surrogate dosimetry in treatments and tumor models where the post-PDT immunological response dominates the observed treatment outcome. This is a particularly important issue, because in the cases where immunology dominates the biological response, neither explicit nor implicit dosimetry measurements may have value. In these cases, searching for surrogate dosimetry measurements that better correlate to the immunological response may be more productive, especially if they are tools widely adopted in clinical practice, such as blood chemistry or imaging.

## 4. Clinical tools available for surrogate dosimetry measures

Surrogates for dosimetry would ideally be predictive of response, perhaps reporting on an important dose-limiting factor of the treatment. Identifying a metric which correlates with, or otherwise represents the dose-limiting factor for treatment response (specific for the PS used and the tumor type treated), is perhaps the key in choosing surrogate measures. The steps in establishing a robust surrogate metric for PDT are outlined in table 2. The measurements need not necessarily directly correlate to an explicit dosimetry parameter, but ideally they would. This would insure that if patient-specific treatment was designed, then the explicit para meters could be systematically altered to improve therapy outcome. Examples of clinically used radiological procedures and clinical chemistry tests are outlined in the next section, with an eye towards future utility in PDT dosimetry.

In photodynamic therapy, the relationship between light energy delivered and actual dose deposition is constantly changing as a function of photosensitizer concentration. Therefore, the abstraction of *pharmacokinetics*—delivery, uptake and clearance of a drug—is considered as a major leverage point when optimizing PDT therapy. Photosensitizer uptake and retention depends on a number of factors within the body: arterial delivery of PS to the vascular bed (arterial input function), excretion rate (clearing PS from the plasma), extravasation to the tissue via gaps in the endothelial cells lining the vasculature, and leakage back into the vasculature from the tissue. Many of these factors can be characterized through dynamic-contrast enhanced imaging or tracer kinetic imaging, highlighting the potential for implicit clinical dosimetry. Tracer kinetic imaging is so-called because it uses contrast-enhancing molecules that ‘trace’ the path taken by a substance dissolved in the blood, in a manner driven by the vascular and tissue kinetics of the body. Acquired time-series of images can be analyzed using a variety of parametric (Kety 1951, Patlak *et al* 1983) and non-parametric models (Cenic *et al* 1999). The most common imaging modalities used in the clinic, and therefore the most likely candidates for dosimetry, are ultrasound, dynamic contrast-enhanced (DCE) computed tomography (CT) and DCE magnetic resonance imaging (MRI). While a handful of recent studies (Jermyn *et al* 2014, Elliott *et al* 2015) have specifically looked at the relationship between kinetic parameters and photosensitizer uptake or efficacy, most of the literature has focused on either treatment monitoring (Xiang *et al* 2007) or image-guidance of specific drug delivery systems (Maeda *et al* 2001). A summary of such approaches is described in table 3.

Ultrasound is one of the most commonly used imaging modalities in the world, and standard ultrasound B-scan images can localize tumors and assess tumor size, but provide little information on microvessel flow or density. Doppler imaging has been used to study tumor blood flow both clinically and experimentally (Okihara *et al* 1999, Peters-Engl *et al* 1999, Goertz *et al* 2002). Contrast-enhanced ultrasound with microbubbles may be particularly useful when evaluating macromolecular PDT delivery systems, such as BPD nanocells, but will exhibit markedly different kinetics than typical contrast agents and smaller drugs (Maeda *et al* 2001). However the drawbacks of US imaging is the obviously low signal-to-noise ratio in the images, and the nonlinearities resulting from this, together with the 2D nature of the image data.

The most readily translatable information for the purpose of PDT dose planning with vascular acting photosensitizers, is derived from vascular activity images from DCE-MRI or DCE-CT (Elliott *et al* 2015). Though not nearly as clinically widespread as single time-point enhancement images, dynamic time-series images can provide a number of key kinetic parameters. Both modalities offer some common information, as well as certain specialized strengths. DCE-MRI provides an assessment of *K*_trans_, the rate of transfer between the intravascular space and the extravascular extracellular space, which is the product of blood flow and extraction fraction leakiness (Sourbron and Buckley 2011). Tissue distribution volume, *v*_*e*_, is also readily available. To date, DCE-MRI has been used extensively to evaluate the response of a tumor to treatment longitudinally and to assess the tumor vasculature in terms of permeability and vascular density, hallmarks of angiogenesis. From a dosimetry perspective, several studies have investigated the use of DCE-MRI parameters to predict responses to targeted therapies in renal cell carcinoma (Rosen and Schnall 2007), dexamethasone (Armitage *et al* 2007) and chemoradiation therapy in rectal cancer (Devries *et al* 2001). DCE-CT can provide similar measurements, although dose-limits can limit accurate characterization of *v*_*e*_. The endogenous-contrast technique of arterial spin labelling (ASL) MRI is attractive because it does not introduce a foreign substance into the patient, and can be used repeatedly without concern for nephrotoxicity. However, it is not used routinely in the clinic, and has only been demonstrated in monitoring brain tumors (Silva *et al* 2000). A major strength of DCE-CT is its ability to characterize first-pass kinetics—blood flow (BF), blood volume (BV), capillary transit time (*T*_c_), extraction fraction (*E*), and permeability-surface area (PS) product—which are arguably easier to quantitate than in DCE-MRI. Most modern clinical scanners are equipped with the necessary algorithm to perform these perfusion CT measurements (Cenic *et al* 1999, Sahani *et al* 2005). Similarly, as technology improvements are realized in C-ARM flat-panel fluoroscopy systems, CT-like imaging is becoming available in operating rooms or interventional radiology environments (Wallace *et al* 2008), and emerging technology provides *in situ* estimates of perfusion parameters (Ganguly *et al* 2010).

The relationship between parameters obtained from these imaging modalities and the true spatial and temporal deposition of drug are complex. For example, the relationship between *K*_trans_ and molecular weight when DCE-MRI is performed using a series of differently sized gadolinium-complex molecules obeys a power law (de Lussanet *et al* 2005). In addition to molecular weight, permeability and retention are influenced by charge, lipid solubility, and propensity for non-specific binding. The latter is the reason why the <1 kDa sized indocyanine green, which bindings strongly with the 66.5 kDa ubiquitous albumin protein, acts as a very large MW vascular tracer and not a small MW permeable tracer. Yet, it is evident from the large number of studies investigating the relationship between angiogenesis, blood volume, microvessel density and permeability that effective radiologic dosimetry will be realized only if imaging parameters can be mapped to the behavior of photosensitizers. Standardized procedures developed to determine the specific relationships between clinical contrast tracers and photosensitizer drugs are needed for large-scale deployment of this dosimetry approach.

Table 3 summarizes the possible contrast imaging methods, all accepted radiological modalities, which could contribute as surrogate imaging tools for dosimetry.

Biopsy and blood collection are standard practices in the workup of most patients. The key issue is the timing of the collection and how this impacts the interpretive value of the data. The data that is routinely available from blood draw are hematocrit, PS fluorescence in the plasma, circulating proteins, bilirubin and tumor mRNA/DNA. These latter three would be direct measures of tumor damage. Hematocrit levels factor into light attenuation in tissue, and PS plasma levels have routinely been sampled from patients to understand patient to patient variability when trying a new photosensitizer. Biopsies are rarely taken from treated tumors, but pre-treatment biopsy is the norm in many sites, and can be used to estimate vascular density and areas, which could predict light delivery issues or drug delivery issues, depending upon which is the dose limiting factor in a treatment. The ease of sampling and the fact that these two measurements are highly integrated into patient workflow are important factors in thinking about these as potential targets for surrogate dosimetry.

## 5. Example of surrogate dosimetry: contrast imaging

Examples of the process of distilling dosimetry to surrogate parameters have been illustrated in PDT of solid cancers, using vascular delivered sensitizers. This is a particularly important model because the impact of treatment is inherently localized by the light penetration out from the fiber delivery location, and that the treatments are inherently limited by drug delivery, light delivery or oxygen delivery, but not often not limited by more than one of these. So this means that there is an opportunity to determine which single parameter most dominates the treatment responses of the therapy and using clinical measurement tools as a surrogate metric for this.

**Figure 3.**
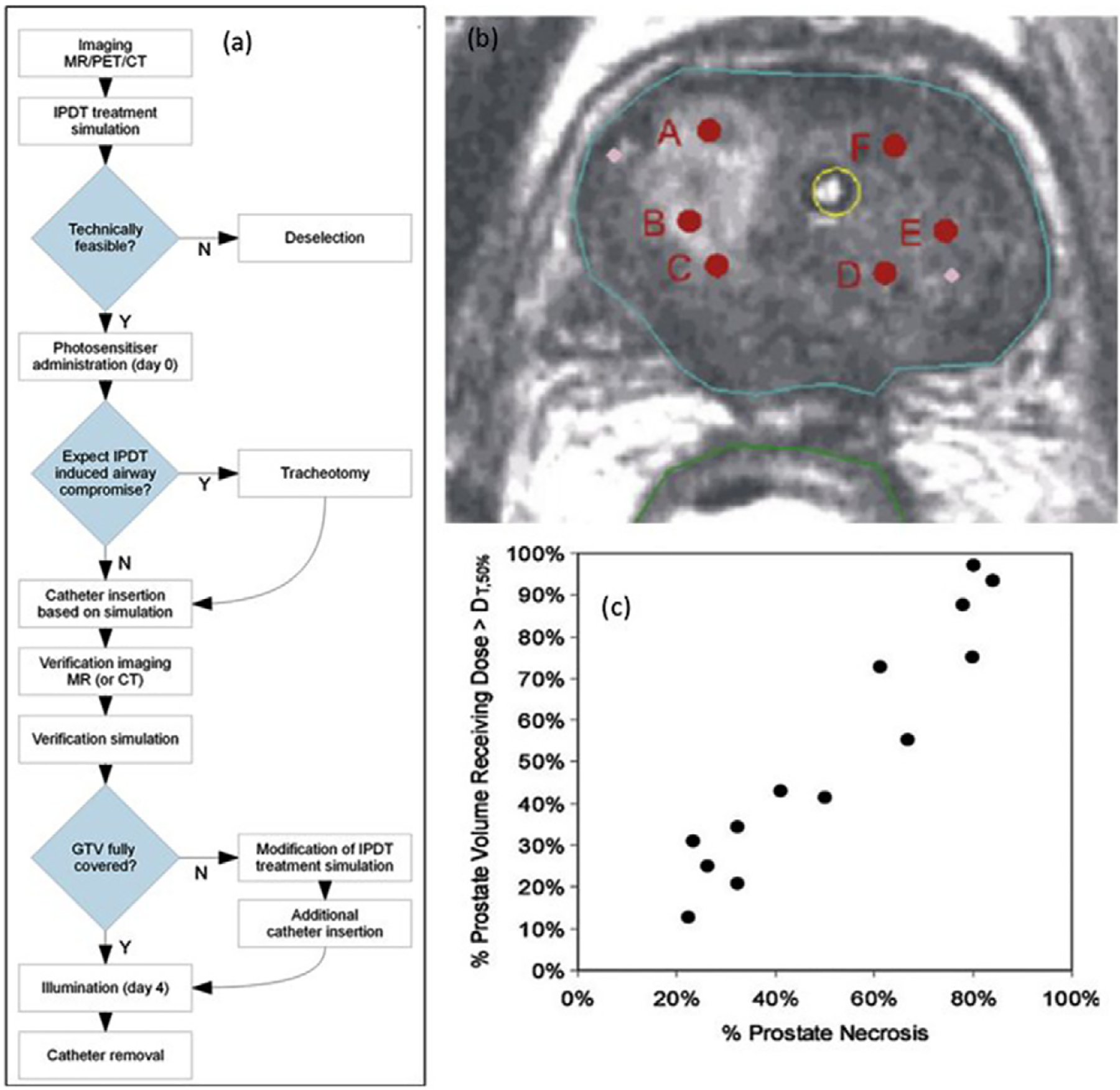
One example of basic use of radiological imaging in dosimetry is outlined in the flow chart of (a) where pre-treatment MRI/PET/CT is used to assess if the treatment plan indicates a technically feasible treatment (reproduced with permission from Karakullukcu *et al* 2013). In prostate treatment planning (b), (reproduced with permission from Davidson *et al* 2009) for Tookad-based PDT, the % induced necrosis was found to be directly correlated to the % volume receiving a light dose over the threshold dose estimate, supporting that light delivery is a dominant factor in treatment efficacy.

An example of the basic use of radiological images to triage subject suitability was demonstrated by Karakullukcu *et al* (2013) in the treatment planning of head and neck cancers with Foscan, where the pre-operative CT or MRI scan was technically feasible for delivery, as illustrated in their flow chart of figure 3(a). This is a basic implementation of this information, allowing assessment of geometry and complete pre-treatment planning with light delivery simulation. In most solid tumor treatments though, interventional imaging during the placement of delivery fibers is done during phase 1 studies. This is completed to verify the placement and probe measurements between fibers can be achieved to verify the light delivery process in real time. This is a straightforward example of how a pre-treatment CT scan could be used for dosimetric value, and reduction of inter-patient response variability by making decisions prior to PDT treatment. Since most clinically implemented PDT (beyond clinical trials) is done without any dosimetry at all, this basic interpretation of imaging is one step towards surrogate dosimetry value.

CT scanning is commonly implemented for post-treatment evaluation in solid tuomrs, because the responses often appear as a hypo-contrast area when the vasculature is ablated by the treatment. Light delivery in prostate cancer treatment has been developed for a number of different photosensitizers and simulation software used with pre-treatment MR or CT scans (Chen and Hetzel 1998, Chen *et al* 2002, Weersink *et al* 2005, Du *et al* 2006, Zhu and Finlay 2006, Johansson *et al* 2007, Li *et al* 2008, Li and Zhu 2008, Davidson *et al* 2009). The work by Davidson *et al* (Davidson *et al* 2009) shown in figures 3(b) and (c) indicates that treatment planning estimation is required, because the volume of necrosis directly correlated to the fraction of the tumor treated to greater than 50% of the threshold dose. While this is not an illustration of pre-measurement planning, it shows that post-PDT scans correlate to the light delivery, which is a challenge to measure in as the treatment is administered. The vascular delivery and fast action of injection and treatment of this photosensitizer suggests that the treatment light delivery would be a direct predictor of response, as was seen. If this trial follows into 2nd phase studies, it may be possible to find surrogates of response which would correlate with treatment outcome, related to the pre-treatment CT scans.

**Figure 4.**
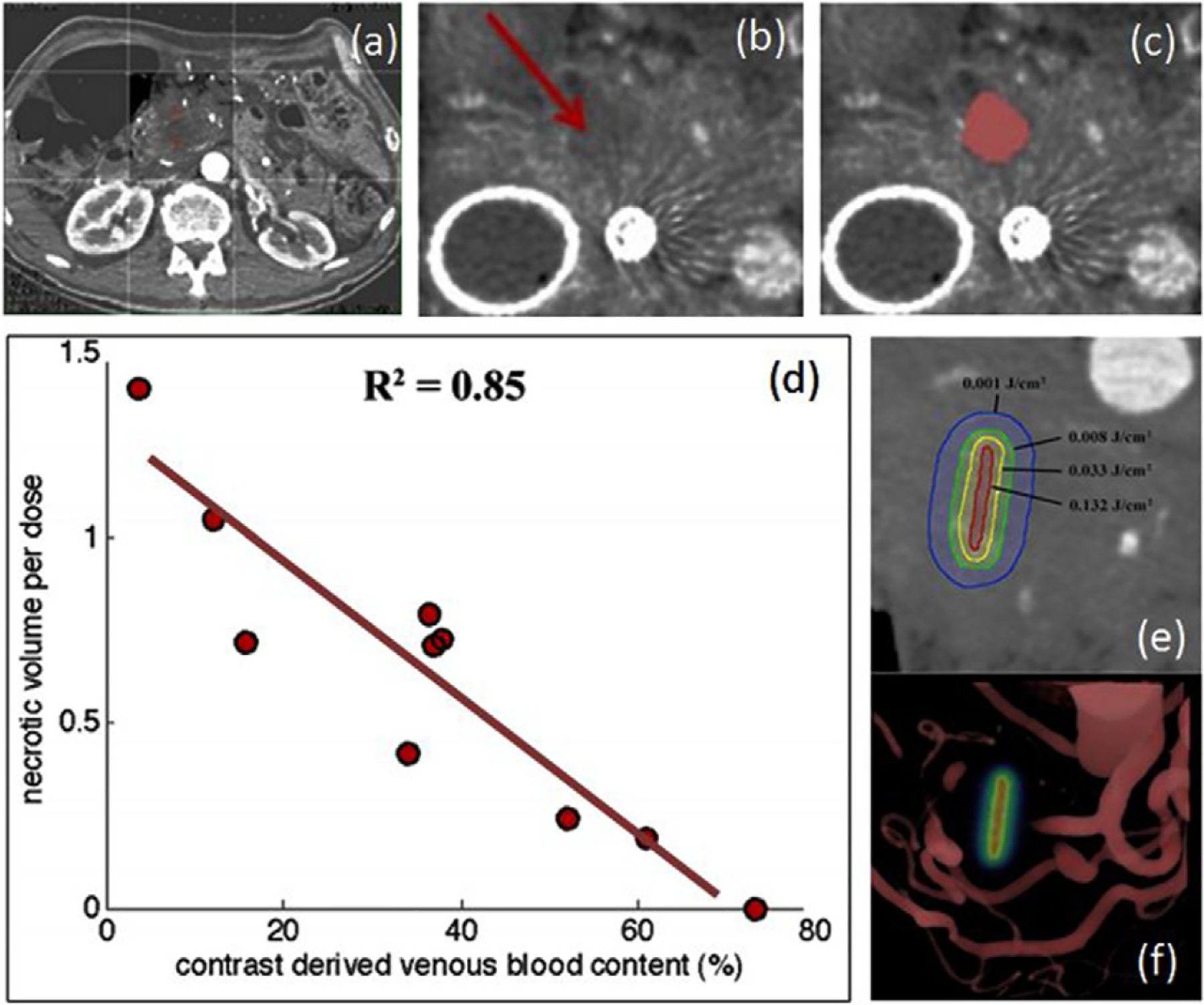
A case from the pancreas cancer treatment clinical trial with verteporfin-PDT is shown (a) with placement of the fiber in the pancreas for light treatment. The PDT-induced lesion sizes were imaged by contrast CT (b), and volumes thresholded to quantify the effect (c). When correlation was tested between the pre-PDT contrast enhancement relative to the necrotic volume per unit dose (volume normalized by the delivered light dose), there was a strong linear correlation (d). This supports the idea that the light dose (e) was limited by the volumes of blood around the treatment fiber, visualized in (f) (reproduced with permission from Huggett *et al* 2014 and Jermyn *et al* 2014).

Following this idea, a new observation was seen in pre-treatment CT scanning in a clinical trial of verteporfin-based PDT in pancreas cancer. The study was carried out with injected dose of 0.4 mg kg^−1^ and a 60–90 min period prior to interstitial irradiation with 690 nm light. It was a safety trial with light dose escalation from 5 to 40 J cm^−1^, where the effect expected would be a mixture of vascular occlusion/ablation and cellular damage. Many photosensitizers used for solid tumor PDT have a mixture of cellular and vascular damage responses, with the vascular damage being the most obvious to measure by contrast CT. In this study, it was found that pre-treatment contrast-CT images could be analyzed for contrast difference values, and that these values were inversely correlated with the PDT-induced lesion volume as measured by the loss of functional CT contrast, as shown in figure 4 (Huggett *et al* 2014, Jermyn *et al* 2014). Because this was a dose escalation trial, with increasing prescribed light doses, and the lesion volumes were generally found to increase with the average prescribed dose but with some inter-subject variability. However, the correlation between pre-treatment CT contrast and final necrotic volume per light dose unit was much stronger. Because the correlation with blood volume contrast was so strong, these results imply that other factors, such as variations in photosensitizer concentration and tissue oxygenation, were relatively less important.

The fact that blood volume was inversely correlated with necrosis indicates that increased blood volume (i.e. contrast) decreases the treatment effect, leading to the hypothesis that attenuation of the light (due to absorption by blood) is the primary limiting factor for the treatment response. This seems plausible and suggests that CT contrast may represent a singular, measurable dosimetry parameter for this type of treatment. Because pre-treatment contrast-CT scans are a standard part of the clinical workup for pancreatic cancer patients, this finding is readily translatable to larger-scale clinical trials of PDT. Of course, this example is one which has been found through retrospective analysis, and needs validation in prospective studies. Additionally, the hypothesis still must be validated, that changes to the treatment plan based upon the contrast-CT data can positively impact lesion volume and therefore patient outcome. However the same study did indicate that across patients there was a clear dose response with increasing light delivery, so it is reasonable to assume that this might be validated in the future study.

One can hypothesize that many solid tumor treatments would behave similarly. Because the goal of PDT using implanted fibers is to treat outwardly from the fiber, to the largest extent possible, light propagation becomes the key dominating factor in the induced lesion volume. This type of surrogate dosimetry should be examined in cases where PDT treatment is being done interstitially and where pre-treatment work up involves a contrast CT scan. Again, the hypothesis that this can positively affect individual dosimetry remains to be validated in prospective clinical trials, but the concept seems as though it could be adopted by a number of photosensitizer and solid tumor treatment situations. Going forward, the proposed ways to implement contrast-CT based surrogate dosimetry is to:

1. Test increasing the light dose delivery in subjects with high contrast enhancement
2. Attempt a multi-delivery treatment in these patients to enhance effect
3. Examine ways to reduce blood in the local region, to minimize light attenuation

These prospective modifications have not been tested clinically yet, but could be plausibly implemented, to reduce inter-subject variation, and yet not get into extensive explicit dosimetry system development and measurement which could curb enthusiasm for the trial.

## 6. Example of reductionist dosimetry: photobleaching

The most direct implementation of a reductionist approach to dosimetry is to follow the lead of implicit dosimetry. Perhaps the most studied approach to this is directly monitoring photobleaching to adjust the light delivery. As shown in a human trial published by Johansson *et al* (Hennig *et al* 2011, Johansson *et al* 2013), the amount of PpIX present and the completeness of the photobleaching from before to after light treatment was a direct indicator of completeness of treatment efficacy. The proposed reduced approach to dosimetrically measuring treatment completion is to sample fluorescence periodically throughout treatment and increase the prescribed light treatment until a suitable bleaching level has been achieved (Hennig *et al* 2011). While adjustment of the light fiber orientation and placement is still routine in PDT treatment planning, the implementation of active dosimetry which is used to individualize treatment is still not conventional, however.

**Figure 5.**
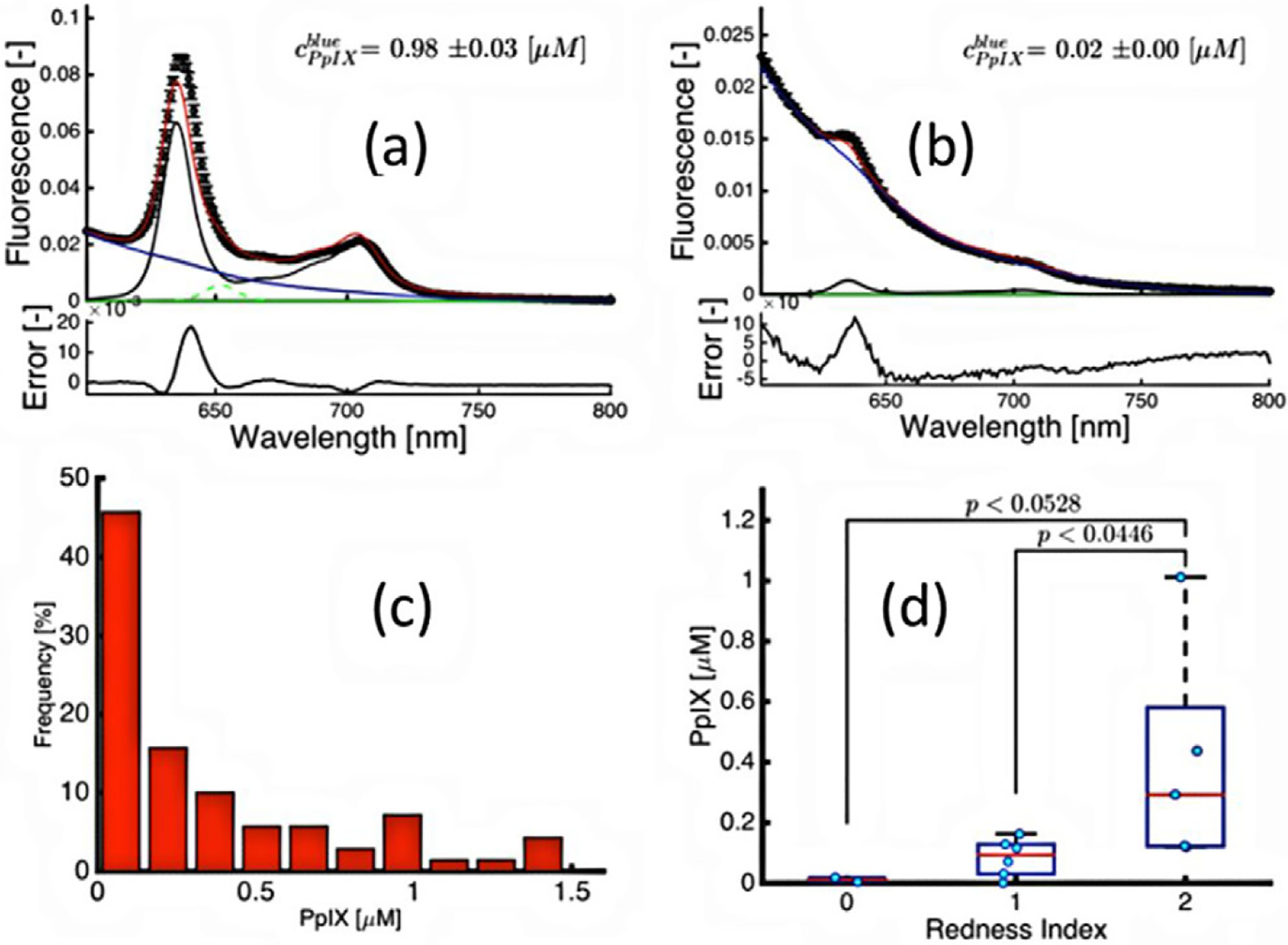
Data from clinical ALA-PDT treatments of AK, showing (a) the strong fluorescence signature of PpIX emission in a responding patient compared with (b) the absence of PpIX fluorescence in a non-responding patient, each fitted for PpIX concentration, inset with fitting error below. In (c), the data histogram of all subjects shows that nearly 40% of patients (*n* = 70) presented very low PpIX concentrations. In (d) the patients binned into 3 groups of erythema scores show significant differences in the PpIX concentration value at the time of treatment (Kanick *et al* 2015).

In dermatology, the same type of distillation or reductionist approach to finding the singular limiting dose parameter has been examined, using ALA-PpIX photobleaching (Van der Veen *et al* 1997, Robinson *et al* 1998, 1999, Robinson *et al* 2000, Warren *et al* 2010). For dermatological applications of PDT, optical measurements can readily be assessed on tissue surfaces. Recently optical measurements were incorporated into a pilot clinical invest igation of ALA-PpIX for AK (Kanick *et al* 2015), as shown in figure 5. A spectroscopic probe system was used to measure fluorescence emission figure 5(a) and decompose the signal into PpIX and background contributions. Measurements before treatment are shown for a subject with high production of PpIX (a), and a subject with low production (b). These measurements could be taken before and after treatment to verify the photobleaching process. However, one of the most important observations from data collected from 70 patients was that the pre-treatment PpIX concentration showed very large inter-patient variability, as seen in the figure 5(c) frequency histogram. The tissue response in the days after treatment as measured by redness index showed direct correlation to the amount of PpIX present at treatment, shown in figure 5(d), suggesting that patient-specific PpIX generation may be a limiting factor in clinical treatments. The data suggest that dosimetric assessment of PpIX generation prior to treatment may identify non-responding patients at the time of treatment, and allow for clinical interventions to improve the probability of successful response. While much attention has been paid to photobleaching, it is quite common that the photobleaching amount correlated to the amount of PpIX present at the start of treatment, and that so the more dominant factor affecting things is simply how much PpIX is produced in each individual lesion. Given the approach to PpIX PDT, it is almost always possible to photobleach away the compound by illuminating until this is achieved. IT may be more important though to alter the delivery of ALA prior to PDT to enhance the production of PpIX as much as is feasible in each subject.

The ultimate implementation of this in a clinically adoptable package, would be to seamlessly integrate fluorescence measurements into the treatment device, as has been demonstrated in a number of research applications. Most recently, a clinical tool having this feature was widely produced (Andrade *et al* 2014), and this could eventually be used to track photobleaching in real time. This integrated package is unique in that it has been integrated into the commercially available devices used in Brazil with widespread distribution (Andrade *et al* 2014), allowing each physician team to understand the likelihood of response in every treatment with minimal extra time required. Unfortunately this is not the case with clinical PDT treatments in most countries, where it is not common to sample PpIX fluorescence at all, much less to follow photobleaching. While a large number of individual studies have demonstrated the interpretive value of fluorescence imaging of PpIX treatment with other measurement devices (Svanberg *et al* 1998, Fischer *et al* 2001, Ericson *et al* 2005), there still is not systematic monitoring of PpIX production in most commonly implemented clinical treatments of PpIX-based PDT. As a result, the heterogeneity of responses in skin treatments is unfortunately high (Wang *et al* 2009); much of this might be corrected with the integration of a simple single dosimetry measurement (Tyrrell *et al* 2010b). Zietouni *et al* (2014) implemented one type of modified treatment, where the light fluence was adjusted to a two-step irradiance proto col, in order to reduce pain and test improved treatment outcome with lower fluence rates. This is one step towards a tailored dose rate delivery, but individualization of the treatment plan would be the next stage in development of a custom treatment planning process.

The next step in this process is perhaps the most critical though to truly see the fruition of dosimetry reduction, which is to carry out prospective trials that test the hypothesis that the prescribed treatment should change in response to pre-treatment measurement of PpIX levels. Since PpIX photobleaches so strongly, the approach of applying more light delivery is not likely to have much value as a dose modification approach in PpIX-based PDT, but rather there would be a need to increase the observed production at the site to be treated. For example, if low sensitizer levels were observed in skin, the possible next step would then be to:

1. Apply more ALA or wait for longer period of time
2. Provide some added method for epithelium removal (tape stripping)
3. Provide occlusion to amplify the penetration of ALA into the skin
4. Modify the biology of the tumor through differentiation therapy approaches

Each of these approaches could have value, and individualization of the treatment process is required to see value in the reduced approach to dosimetry.

## 7. Summary

For pre-clinical work and even for phase I human trials, both explicit and implicit dosimetry are needed to gain the maximum mechanistic understanding from the data, and to test for correlations to treatment outcome. This information is central to the development process, because each tumor type and photosensitizer type has unique characteristics. Unfortunately, the number of important parameters to characterize exceeds six (tables 4 and 5), and quickly reaches a large number of measurements, especially if temporal kinetic changes during treatment are considered (Wilson *et al* 1997, 2010, Zhu 2012). However, it is critical for the field of PDT that this large parameter space not become the cause of failed clinical trials nor physician refusal to adopt PDT in their practices. Furthermore, recent information has shown that clinical outcomes may not be linearly related to factors associated with delivered dose (van Duijnhoven *et al* 2003, Kabingu *et al* 2009, Kammerer *et al* 2011), due to a number of nonlinear biological factors.

Therefore, as PDT treatments move into Phase II trials, it is important to identify (for a given tumor type and photosensitizer) the one or two key factors that truly limit the dose delivery, and that best correlate to the treatment response. The key challenge for dosimetry is identifying these parameters and choosing clinically acceptable tools capable of measuring those parameters. Ultimately, these measurement modalities will fall within the categories of explicit dosimetry, implicit dosimetry, or surrogate dosimetry. In the case of surrogate dosimetry, some situations will lend themselves to standard clinical practice, which could be ideal for improving physician/patient acceptance of the procedure.

Distillation of dosimetry measurements to the essential factors that dominate the treatment response, along with identification of clinically acceptable surrogates, will be essential to future successes of PDT. While maximal information is always better for scientific understanding, minimal overhead is desired for clinical and commercial success. Finding the proper balance between these competing goals represents an important challenge for the future of PDT as a clinical oncology therapy modality.

## Figures and Tables

**Table 1.** The factors affecting photosensitizer location and effect are tabulated.

Factors affecting Photosensitizer	Example	References
Variable production of PPIX	Actinic keratosis variation	(Ericson *et al* 2004, McLoone *et al* 2008, Wiegell *et al* 2011, Wiegell *et al* 2012)
	Penetration of ALA	
	Keratin layers presence	
	Variability in skin occlusion use	
	Variability in application times	
Vascular versus cellular location of PS from time after IV injection	Vascular delivery required for solid tumors	(Fingar *et al* 1992b, Schmidt-Erfurth *et al* 1995, Chen *et al* 2005a, Chen *et al* 2006)
	Transvascular permeability variations	
Intracellular redistribution in cells	Not an issue for all sensitizers but well known for lipid localizing agents (AlPcS_2_) and PpIX	(Strauss *et al* 1995, Fingar, 1996, Georgakoudi and Foster 1998a, Fingar *et al* 1999, Kessel and Poretz 2000, Krieg *et al* 2003, Chen *et al* 2005a, Osaki *et al* 2006, Yu *et al* 2006, Sailer *et al* 2007, Trachtenberg *et al* 2007, Woodhams *et al* 2010)
	Well documented for most PS	

**Table 2.** The clinical trial phases are tabulated along with the stages of dosimetry appropriate for each.

Trial stage	Dosimetry stage
Pre-clinical	Vary dose factors systematically to determine which dominate response outcome Test surrogate measurements for correlation to response Prospectively test delivery verification
Phase 1	Verify/test limiting factors in dose escalation trial Verify/test measurements correlating to dose limiting factor
Phase 2	Perform surrogate measurement Deliver therapy Assess outcome Verify that surrogate measure correlated to outcome Test that changes to dose delivery can improve outcome & that this is predicted by the surrogate dose measurement
Phase 3	Perform surrogate measurement Design dose delivery based upon measurement Deliver customized therapy plan Assess outcome

**Table 3.** Common modalities, their sources of contrast and the parameters which they report.

Modality	Contrast mechanism	Routine parameters	Advanced parameters	Disadvantages	Refs
Ultrasound	Tissue interfaces, red blood cells, microbubbles	Tumor shape, location, density	Perfusion, velocity, elastography, stiffness & matrix	Operator dependent, quantification difficult	(Okihara *et al* 1999, Peters-Engl *et al* 1999, Maeda *et al* 2001, Goertz *et al* 2002)
DCE-MRI	Gadolinium	*T*_1_*, T*_2_, pre/post difference, *K*_trans_, *v*_e_	Blood flow & volume, vascular and tissue *V*_d_, distribution of transit times		(Devries *et al* 2001, Armitage *et al* 2007, Rosen and Schnall 2007)
ASL-MRI	Spin-labeled water	None	Blood flow	Limited mainly to brain imaging	(Silva *et al* 2000)
X-ray C-arm	Iodine	Location, blood flow visual, contrast enhancement patterns & changes	Estimated flow rates	Ionizing radiation	(Wallace *et al* 2008, Ganguly *et al* 2010)
			Estimated arterial/venous values		
DCE-CT	Iodine	CT number, arterial & venous temporal contrast enhancement	Blood flow, volume, mean transit time, extraction, Permeability surface, *k*_ep_	Ionizing radiation, motion artifacts for thorax	(Cenic *et al* 1999, Sahani *et al* 2005)

**Table 4.** Explicit dosimetry measurements are the key factors in dose, including: (1) photosensitizer, (2) light and (3) oxygen.

Dose parameter	Prescribed	Minimal sampling	Verification points	2D data	3D data	Refs
Photosensitizer	Injected dose	Plasma sampling	Tissue point absorbance/fluorescence	Tissue surface imaging fluorescence	Diffuse fluorescence tomography	(Braichotte *et al* 1996, Farrell *et al* 1998a, Boere *et al* 2003, Yang *et al* 2003, Valentine *et al* 2013, Kanick *et al* 2014, Mallidi *et al* 2014)
Light	Delivered power/area	Intra-organ measures	Surface or diffuser probes	Interstitial tissue diffuser probes	Diffuse tomography	(Grossweiner, 1986, Powers and Brown, 1986, Werkhaven *et al* 1986, Wilson *et al* 1986, Grossweiner *et al* 1987, Potter *et al* 1987, Star *et al* 1987, Arnfield *et al* 1989, D’Hallewin *et al* 1992, Foster and Gao, 1992, Marijnissen *et al* 1993, Heier *et al* 1995, Beyer, 1996, Braichotte *et al* 1996, Svaasand *et al* 1996, Tromberg *et al* 1996, Bays *et al* 1997, Wilson *et al* 1997, Lilge *et al* 1998, Tan *et al* 1999, Johansson *et al* 2002, van Veen *et al* 2002, Boere *et al* 2003, Radu *et al* 2003, Dickey *et al* 2004, van Veen *et al* 2006, Zhu and Finlay, 2006, Johansson *et al* 2007, Swartling *et al* 2010, Zhu 2012)
Oxygen	Blood SO_2_	Blood flow monitoring / optical imaging	Point sampling of blood flow, SaO_2_ or pO_2_	Needle electrode tracks / structured light imaging	Diffuse SO_2_ tomography	(Tromberg *et al* 1990, Foster and Gao, 1992, Georgakoudi and Foster 1998, 1998b, Kelleher *et al* 2004, Zhu *et al* 2005, Woodhams *et al* 2007, Jarvi *et al* 2011, Weston and Patterson 2013)

*Note*: These are listed with the ways in which they have been shown to be quantified in mechanistic and translational studies.

**Table 5.** Methods for implicit dosimetry are outlined with values measured and some of the limitations of each technique.

Method	Value	Limitations	Refs
Photosensitizer photobleaching	Direct sampling of PS activated High signal to noise	Limited to surface sampling, point probe measurements or potentially diffuse tomography potential disconnect between ‘bulk’ tissue and microscopic localization of PS	(Georgakoudi *et al* 1997, Farrell *et al* 1998a, Iinuma *et al* 1999, Boere *et al* 2003, Sheng *et al* 2007, Pogue *et al* 2008, Jarvi *et al* 2012)
Photosensitizer excited state lifetime measurement	Direct sampling of PS and its instantaneous interactions with oxygen or other molecules	Specific to oxygen sensitive excited states Low signal to noise	(Redmond *et al* 1994, Aveline *et al* 1995, 1998, Pogue *et al* 2001b)
Singlet oxygen phosphorescence	Direct end product of PDT dose	Limited to surface sampling or point probe measurements. Low signal to noise	(Douzou, 1972, Niedre *et al* 2005, Jarvi *et al* 2006, Laubach *et al* 2008, Lee *et al* 2011, Jarvi *et al* 2012, Mallidi *et al* 2014)
Free radical damage to tissue	Membrane damage Necrosis morphology Enzyme & blood component invasion	Restricted to research Requires use of exogenous probes in some cases	(Mitra *et al* 2013)

## References

[R1] Agostinis P 2011 Photodynamic therapy of cancer: an update CA Cancer J. Clin 61 250–81 21617154 10.3322/caac.20114 PMC3209659

[R2] Akilov OE, Kosaka S, Maytin EV and Hasan T 2008 Prospects for the use of differentiation-modulating agents as adjuvant of photodynamic therapy for proliferative dermatoses J. Dermatol 35 197–205 18419676 10.1111/j.1346-8138.2008.00445.x

[R3] Anand S, Hasan T and Maytin EV 2013 Mechanism of differentiation-enhanced photodynamic therapy for cancer: upregulation of coproporphyrinogen oxidase by C/EBP transcription factors Mol. Cancer Ther 12 1638–50 23686770 10.1158/1535-7163.MCT-13-0047 PMC3743443

[R4] Anand S, Wilson C, Hasan T and Maytin EV 2011 Vitamin D3 enhances the apoptotic response of epithelial tumors to aminolevulinate-based photodynamic therapy Cancer Res. 71 6040–50 21807844 10.1158/0008-5472.CAN-11-0805 PMC3360482

[R5] Anbil S, Rizvi I, Celli JP, Alagic N, Pogue BW and Hasan T 2013 Impact of treatment response metrics on photodynamic therapy planning and outcomes in a 3D model of ovarian cancer J. Biomed. Opt 18 098004 24802230 10.1117/1.JBO.18.9.098004 PMC3783041

[R6] Andrade CT, Vollet-Filho JD, Salvio AG, Bagnato VS and Kurachi C 2014 Identification of skin lesions through aminolaevulinic acid-mediated photodynamic detection Photodiagn. Photodyn. Ther 11 409–15 10.1016/j.pdpdt.2014.05.006 24892509

[R7] Armitage PA, Schwindack C, Bastin ME and Whittle IR 2007 Quantitative assessment of intracranial tumor response to dexamethasone using diffusion, perfusion and permeability magnetic resonance imaging Magn. Reson. Imaging 25 303–10 17371718 10.1016/j.mri.2006.09.002

[R8] Arnfield MR, Tulip J, Chetner M and McPhee MS 1989 Optical dosimetry for interstitial photodynamic therapy Med. Phys 16 602–8 2770633 10.1118/1.596361

[R9] Aveline BM, Hasan T and Redmond RW 1995 The effects of aggregation, protein binding and cellular incorporation on the photophysical properties of benzoporphyrin derivative monoacid ring A (BPDMA) J. Photochem. Photobiol. B 30 161–9 8558368 10.1016/1011-1344(95)07174-z

[R10] Aveline BM, Sattler RM and Redmond RW 1998 Environmental effects on cellular photosensitization: correlation of phototoxicity mechanism with transient absorption spectroscopy measurements Photochem. Photobiol 68 51–62 9679451

[R11] Bajgar R, Kolarova H, Bolek L, Binder S, Pizova K and Hanakova A 2014 High oxygen partial pressure increases photodynamic effect on HeLa cell lines in the presence of chloraluminium phthalocyanine Anticancer Res. 34 4095–9 25075034

[R12] Bays R, Wagnieres G, Robert D, Braichotte D, Savary JF, Monnier P and van den Bergh H 1997 Light dosimetry for photodynamic therapy in the esophagus Lasers Surg. Med 20 290–303 9138258 10.1002/(sici)1096-9101(1997)20:3<290::aid-lsm8>3.0.co;2-l

[R13] Beyer W 1996 Systems for light application and dosimetry in photodynamic therapy J. Photochem. Photobiol. B 36 153–6 9002252 10.1016/s1011-1344(96)07363-0

[R14] Bicher HI, Hetzel FW, Sandhu TS, Frinak S, Vaupel P, O’Hara MD and O’Brien T 1980 Effects of hyperthermia on normal and tumor microenvironment Radiology 137 523–30 7433686 10.1148/radiology.137.2.7433686

[R15] Blake E, Allen J and Curnow A 2011 An *in vitro* comparison of the effects of the iron-chelating agents, CP94 and dexrazoxane, on protoporphyrin IX accumulation for photodynamic therapy and/or fluorescence guided resection Photochem. Photobiol 87 1419–26 21834866 10.1111/j.1751-1097.2011.00985.x

[R16] Boere IA, Robinson DJ, de Bruijn HS, van den Boogert J, Tilanus HW, Sterenborg HJ and de Bruin RW 2003 Monitoring *in situ* dosimetry and protoporphyrin IX fluorescence photobleaching in the normal rat esophagus during 5-aminolevulinic acid photodynamic therapy Photochem. Photobiol 78 271–7 14556314 10.1562/0031-8655(2003)078<0271:misdap>2.0.co;2

[R17] Brackett CM and Gollnick SO 2011 Photodynamic therapy enhancement of anti-tumor immunity Photochem. Photobiol. Sci 10 649–52 21253659 10.1039/c0pp00354a PMC3197776

[R18] Braichotte DR, Savary JF, Monnier P and van den Bergh HE 1996 Optimizing light dosimetry in photodynamic therapy of early stage carcinomas of the esophagus using fluorescence spectroscopy Lasers Surg. Med 19 340–6 8923430 10.1002/(SICI)1096-9101(1996)19:3<340::AID-LSM10>3.0.CO;2-8

[R19] Canti G, Lattuada D, Nicolin A, Taroni P, Valentini G and Cubeddu R 1994 Antitumor immunity induced by photodynamic therapy with aluminum disulfonated phthalocyanines and laser light Anticancer Drugs 5 443–7 7949249 10.1097/00001813-199408000-00009

[R20] Celli JP, Spring BQ, Rizvi I, Evans CL, Samkoe KS, Verma S, Pogue BW and Hasan T 2010 Imaging and photodynamic therapy: mechanisms, monitoring, and optimization Chem. Rev 110 2795–838 20353192 10.1021/cr900300p PMC2896821

[R21] Cenic A, Nabavi DG, Craen RA, Gelb AW and Lee TY 1999 Dynamic CT measurement of cerebral blood flow: a validation study AJNR Am. J. Neuroradiol 20 63–73 9974059

[R22] Chen B, Crane C, He C, Gondek D, Agharkar P, Savellano MD, Hoopes PJ and Pogue BW 2008 Disparity between prostate tumor interior versus peripheral vasculature in response to verteporfin-mediated vascular-targeting therapy Int. J. Cancer 123 695–701 18498134 10.1002/ijc.23538

[R23] Chen B, Pogue BW, Goodwin IA, O’Hara JA, Wilmot CM, Hutchins JE, Hoopes PJ and Hasan T 2003 Blood flow dynamics after photodynamic therapy with verteporfin in the RIF-1 tumor Radiat. Res 160 452–9 12968929 10.1667/RR3059

[R24] Chen B, Pogue BW, Hoopes PJ and Hasan T 2005a Combining vascular and cellular targeting regimens enhances the efficacy of photodynamic therapy Int. J. Radiat. Oncol. Biol. Phys 61 1216–26 15752904 10.1016/j.ijrobp.2004.08.006

[R25] Chen B, Pogue BW, Luna JM, Hardman RL, Hoopes PJ and Hasan T 2006 Tumor vascular permeabilization by vascular-targeting photosensitization: effects, mechanism, and therapeutic implications Clin. Cancer Res 12 917–23 16467106 10.1158/1078-0432.CCR-05-1673

[R26] Chen B, Pogue BW, Zhou X, O’Hara JA, Solban N, Demidenko E, Hoopes PJ and Hasan T 2005b Effect of tumor host microenvironment on photodynamic therapy in a rat prostate tumor model Clin. Cancer Res 11 720–7 15701861

[R27] Chen JY, Mak NK, Yow CM, Fung MC, Chiu LC, Leung WN and Cheung NH 2000 The binding characteristics and intracellular localization of temoporfin (mTHPC) in myeloid leukemia cells: phototoxicity and mitochondrial damage Photochem. Photobiol 72 541–7 11045727 10.1562/0031-8655(2000)072<0541:tbcail>2.0.co;2

[R28] Chen Q and Hetzel FW 1998 Laser dosimetry studies in the prostate J. Clin. Laser Med. Surg 16 9–12 9728124 10.1089/clm.1998.16.9

[R29] Chen Q, Huang Z, Luck D, Beckers J, Brun PH, Wilson BC, Scherz A, Salomon Y and Hetzel FW 2002 Preclinical studies in normal canine prostate of a novel palladium-bacteriopheophorbide (WST09) photosensitizer for photodynamic therapy of prostate cancers Photochem. Photobiol 76 438–45 12405153 10.1562/0031-8655(2002)076<0438:PSINCP>2.0.CO;2

[R30] Chen Z, Milner TE, Wang X, Srinivas S and Nelson JS 1998 Optical Doppler tomography: imaging *in vivo* blood flow dynamics following pharmacological intervention and photodynamic therapy Photochem. Photobiol 67 56–60 9477766

[R31] Choudry K, Brooke RC, Farrar W and Rhodes LE 2003 The effect of an iron chelating agent on protoporphyrin IX levels and phototoxicity in topical 5-aminolaevulinic acid photodynamic therapy Br. J. Dermatol 149 124–30 12890205 10.1046/j.1365-2133.2003.05351.x

[R32] Cottrell WJ, Paquette AD, Keymel KR, Foster TH and Oseroff AR 2008 Irradiance-dependent photobleaching and pain in delta-aminolevulinic acid-photodynamic therapy of superficial basal cell carcinomas Clin. Cancer Res 14 4475–83 18628462 10.1158/1078-0432.CCR-07-5199 PMC2810858

[R33] Coutier S, Mitra S, Bezdetnaya LN, Parache RM, Georgakoudi I, Foster TH and Guillemin F 2001 Effects of fluence rate on cell survival and photobleaching in meta-tetra-(hydroxyphenyl)chlorin-photosensitized Colo 26 multicell tumor spheroids Photochem. Photobiol 73 297–303 11281027 10.1562/0031-8655(2001)073<0297:EOFROC>2.0.CO;2

[R34] Cramers P, Ruevekamp M, Oppelaar H, Dalesio O, Baas P and Stewart FA 2003 Foscan uptake and tissue distribution in relation to photodynamic efficacy Br. J. Cancer 88 283–90 12610515 10.1038/sj.bjc.6600682 PMC2377038

[R35] Cubeddu R, Pifferi A, Taroni P, Torricelli A, Valentini G, Comelli D, D’Andrea C, Angelini V and Canti G 2000 Fluorescence imaging during photodynamic therapy of experimental tumors in mice sensitized with disulfonated aluminum phthalocyanine Photochem. Photobiol 72 690–5 11107856 10.1562/0031-8655(2000)072<0690:fidpto>2.0.co;2

[R36] Curnow A, McIlroy BW, Postle-Hacon MJ, Porter JB, MacRobert AJ and Bown SG 1998 Enhancement of 5-aminolaevulinic acid-induced photodynamic therapy in normal rat colon using hydroxypyridinone iron-chelating agents Br. J. Cancer 78 1278–82 9823966 10.1038/bjc.1998.671 PMC2063198

[R37] D’Hallewin MA, Baert L, Marijnissen JP and Star WM 1992 Whole bladder wall photodynamic therapy with *in situ* light dosimetry for carcinoma *in situ* of the bladder J. Urol 148 1152–5 1404627 10.1016/s0022-5347(17)36846-5

[R38] Davidson SR 2009 Treatment planning and dose analysis for interstitial photodynamic therapy of prostate cancer Phys. Med. Biol 54 2293–313 19305043 10.1088/0031-9155/54/8/003

[R39] De Goeij AF, Ververgaert PH and Steveninck JV 1975 Photodynamic effects of protoporphyrin on the architecture of erythrocyte membranes in protoporphyria and in normal red blood cells Clin Chim. Acta 62 287–92 1149291 10.1016/0009-8981(75)90238-7

[R40] de Lussanet QG, Langereis S, Beets-Tan RG, van Genderen MH, Griffioen AW, van Engelshoven JM and Backes WH 2005 Dynamic contrast-enhanced MR imaging kinetic parameters and molecular weight of dendritic contrast agents in tumor angiogenesis in mice Radiology 235 65–72 15731376 10.1148/radiol.2351040411

[R41] de Visscher SA, Kascakova S, de Bruijn HS, van den Heuvel AP, Amelink A, Sterenborg HJ, Robinson DJ, Roodenburg JL and Witjes MJ 2011 Fluorescence localization and kinetics of mTHPC and liposomal formulations of mTHPC in the window-chamber tumor model Lasers Surg. Med 43 528–36 21761424 10.1002/lsm.21082

[R42] Devries AF, Griebel J, Kremser C, Judmaier W, Gneiting T, Kreczy A, Ofner D, Pfeiffer KP, Brix G and Lukas P 2001 Tumor microcirculation evaluated by dynamic magnetic resonance imaging predicts therapy outcome for primary rectal carcinoma Cancer Res. 61 2513–6 11289123

[R43] Dickey DJ, Partridge K, Moore RB and Tulip J 2004 Light dosimetry for multiple cylindrical diffusing sources for use in photodynamic therapy Phys. Med. Biol 49 3197–208 15357192 10.1088/0031-9155/49/14/013

[R44] Dognitz N, Salomon D, Zellweger M, Ballini JP, Gabrecht T, Lange N, van den Bergh H and Wagnieres G 2008 Comparison of ALA- and ALA hexyl-ester-induced PpIX depth distribution in human skin carcinoma J. Photochem. Photobiol. B 93 140–8 18818091 10.1016/j.jphotobiol.2008.07.012

[R45] Dougherty TJ, Gomer CJ, Henderson BW, Jori G, Kessel D, Korbelik M, Moan J and Peng Q 1998 Photodynamic therapy J. Natl Cancer Inst 90 889–905 9637138 10.1093/jnci/90.12.889 PMC4592754

[R46] Douzou P 1972 Singlet oxygen as oxidizing intermediate and photodynamic action Res. Prog. Org. Biol. Med. Chem 3 37–47 4681319

[R47] Du KL, Mick R, Busch TM, Zhu TC, Finlay JC, Yu G, Yodh AG, Malkowicz SB, Smith D, Whittington R, Stripp D and Hahn SM 2006 Preliminary results of interstitial motexafin lutetium-mediated PDT for prostate cancer Lasers Surg. Med 38 427–34 16788929 10.1002/lsm.20341

[R48] Edrei R and Kimel S 1999 Oxygen depletion during *in vitro* photodynamic therapy: structure-activity relationships of sulfonated aluminum phthalocyanines J. Photochem. Photobiol. B 50 197–203 10577051 10.1016/S1011-1344(99)00092-5

[R49] Elliott JT, Samkoe KS, Gunn JR, Stewart EE, Gardner TB, Tichauer KM, Lee TY, Hoopes PJ, Pereira SP, Hasan T and Pogue BW 2015 Perfusion CT estimates photosensitizer uptake and biodistribution in a rabbit orthotopic pancreatic cancer model: a pilot study Acad. Radiol10.1016/j.acra.2014.12.014 PMC4395543 25683500

[R50] Ericson MB, Sandberg C, Stenquist B, Gudmundson F, Karlsson M, Ros AM, Rosen A, Larko O, Wennberg AM and Rosdahl I 2004 Photodynamic therapy of actinic keratosis at varying fluence rates: assessment of photobleaching, pain and primary clinical outcome Br. J. Dermatol 151 1204–12 15606516 10.1111/j.1365-2133.2004.06211.x

[R51] Ericson MB, Uhre J, Strandeberg C, Stenquist B, Larko O, Wennberg AM and Rosen A 2005 Bispectral fluorescence imaging combined with texture analysis and linear discrimination for correlation with histopathologic extent of basal cell carcinoma J. Biomed. Opt 10 034009 16229653 10.1117/1.1925650

[R52] Evans CL, Abu-Yousif AO, Park YJ, Klein OJ, Celli JP, Rizvi I, Zheng X and Hasan T 2011 Killing hypoxic cell populations in a 3D tumor model with EtNBS-PDT PLoS One 6 e23434 21876751 10.1371/journal.pone.0023434 PMC3158086

[R53] Farrell TJ, Hawkes RP, Patterson MS and Wilson BC 1998a Modeling of photosensitizer fluorescence emission and photobleaching for photodynamic therapy dosimetry Appl. Opt 37 7168–83 18301543 10.1364/ao.37.007168

[R54] Farrell TJ, Wilson BC, Patterson MS and Olivo MC 1998b Comparison of the *in vivo* photodynamic threshold dose for photofrin, mono- and tetrasulfonated aluminum phthalocyanine using a rat liver model Photochem. Photobiol 68 394–9 9747595

[R55] Fingar VH 1996 Vascular effects of photodynamic therapy J. Clin. Laser Med. Surg 14 323–8 9612199 10.1089/clm.1996.14.323

[R56] Fingar VH, Kik PK, Haydon PS, Cerrito PB, Tseng M, Abang E and Wieman TJ 1999 Analysis of acute vascular damage after photodynamic therapy using benzoporphyrin derivative (BPD) Br. J. Cancer 79 1702–8 10206280 10.1038/sj.bjc.6690271 PMC2362794

[R57] Fingar VH, Mang TS and Henderson BW 1988 Modification of photodynamic therapy-induced hypoxia by fluosol-DA (20%) and carbogen breathing in mice Cancer Res. 48 3350–4 3130983

[R58] Fingar VH, Potter WR and Henderson BW 1987 Drug and light dose dependence of photodynamic therapy: a study of tumor cell clonogenicity and histologic changes Photochem. Photobiol 45 643–50 2955431 10.1111/j.1751-1097.1987.tb07392.x

[R59] Fingar VH, Wieman TJ, Park YJ and Henderson BW 1992a Implications of a pre-existing tumor hypoxic fraction on photodynamic therapy J. Surg. Res 53 524–8 1434604 10.1016/0022-4804(92)90101-5

[R60] Fingar VH, Wieman TJ, Wiehle SA and Cerrito PB 1992b The role of microvascular damage in photodynamic therapy: the effect of treatment on vessel constriction, permeability, and leukocyte adhesion Cancer Res. 52 4914–21 1387584

[R61] Fischer F, Dickson EF, Kennedy JC and Pottier RH 2001 An affordable, portable fluorescence imaging device for skin lesion detection using a dual wavelength approach for image contrast enhancement and aminolaevulinic acid-induced protoporphyrin IX. Part II. *In vivo* testing Lasers Med. Sci 16 207–12 11482819 10.1007/pl00011356

[R62] Flynn BP, DSouza AV, Kanick SC, Davis SC and Pogue BW 2013 White light-informed optical properties improve ultrasound-guided fluorescence tomography of photoactive protoporphyrin IX J Biomed. Opt 18 046008 23584445 10.1117/1.JBO.18.4.046008 PMC3639786

[R63] Foster TH and Gao L 1992 Dosimetry in photodynamic therapy: oxygen and the critical importance of capillary density Radiat. Res 130 379–83 1594766 10.2307/3578385

[R64] Friedberg JS, Culligan MJ, Mick R, Stevenson J, Hahn SM, Sterman D, Punekar S, Glatstein E and Cengel K 2012 Radical pleurectomy and intraoperative photodynamic therapy for malignant pleural mesothelioma Ann. Thorac. Surg 93 1658–65 discussion 65–7 22541196 10.1016/j.athoracsur.2012.02.009 PMC4394024

[R65] Ganguly A, Fieselmann A, Marks M, Rosenberg J, Boese J, Deuerling-Zheng Y, Straka M, Zaharchuk G, Bammer R and Fahrig R 2010 Cerebral CT perfusion using an interventional C-arm imaging system: cerebral blood flow measurements AJNR Am. J. Neuroradiol 32 1525–31 10.3174/ajnr.A2518 PMC3171629 21757522

[R66] Georgakoudi I and Foster TH 1998 Singlet oxygen-versus nonsinglet oxygen-mediated mechanisms of sensitizer photobleaching and their effects on photodynamic dosimetry Photochem. Photobiol 67 612–25 9648527

[R67] Georgakoudi I and Foster TH 1998a Effects of the subcellular redistribution of two nile blue derivatives on photodynamic oxygen consumption Photochem. Photobiol 68 115–22 9679457

[R68] Georgakoudi I and Foster TH 1998b Singlet oxygen-versus nonsinglet oxygen-mediated mechanisms of sensitizer photobleaching and their effects on photodynamic dosimetry Photochem. Photobiol 67 612–25 9648527

[R69] Georgakoudi I, Nichols MG and Foster TH 1997 The mechanism of Photofrin photobleaching and its consequences for photodynamic dosimetry Photochem. Photobiol 65 135–44 9066293 10.1111/j.1751-1097.1997.tb01889.x

[R70] Gerritsen MJ, Smits T, Kleinpenning MM, van de Kerkhof PC and van Erp PE 2009 Pretreatment to enhance protoporphyrin IX accumulation in photodynamic therapy Dermatology 218 193–202 19077380 10.1159/000183753

[R71] Glanzmann T, Hadjur C, Zellweger M, Grosiean P, Forrer M, Ballini JP, Monnier P, van den Bergh H, Lim CK and Wagnieres G 1998 Pharmacokinetics of tetra(m-hydroxyphenyl)chlorin in human plasma and individualized light dosimetry in photodynamic therapy Photochem. Photobiol 67 596–602 9613244

[R72] Goertz DE, Yu JL, Kerbel RS, Burns PN and Foster FS 2002 High-frequency Doppler ultrasound monitors the effects of antivascular therapy on tumor blood flow Cancer Res. 62 6371–5 12438217

[R73] Gollnick SO 2012 Photodynamic therapy and antitumor immunity J. Natl Compr. Cancer Netw 10 S40–3 10.6004/jnccn.2012.0173 PMC3683987 23055214

[R74] Grossweiner LI 1986 Optical dosimetry in photodynamic therapy Lasers Surg. Med 6 462–6 2949127 10.1002/lsm.1900060508

[R75] Grossweiner LI, Hill JH and Lobraico RV 1987 Photodynamic therapy of head and neck squamous cell carcinoma: optical dosimetry and clinical trial Photochem. Photobiol 46 911–7 3327063 10.1111/j.1751-1097.1987.tb04868.x

[R76] Hammer-Wilson MJ, Akian L, Espinoza J, Kimel S and Berns MW 1999 Photodynamic parameters in the chick chorioallantoic membrane (CAM) bioassay for topically applied photosensitizers J. Photochem. Photobiol B 53 44–52 10672528 10.1016/s1011-1344(99)00124-4

[R77] Heier SK, Rothman KA, Heier LM and Rosenthal WS 1995 Photodynamic therapy for obstructing esophageal cancer: light dosimetry and randomized comparison with Nd:YAG laser therapy Gastroenterology 109 63–72 7541003 10.1016/0016-5085(95)90269-4

[R78] Henderson BW and Fingar VH 1987 Relationship of tumor hypoxia and response to photodynamic treatment in an experimental mouse tumor Cancer. Res 47 3110–4 3581062

[R79] Henderson BW and Fingar VH 1989 Oxygen limitation of direct tumor cell kill during photodynamic treatment of a murine tumor model Photochem. Photobiol 49 299–304 2525260 10.1111/j.1751-1097.1989.tb04110.x

[R80] Henderson BW, Sitnik-Busch TM and Vaughan LA 1999 Potentiation of photodynamic therapy antitumor activity in mice by nitric oxide synthase inhibition is fluence rate dependent Photochem. Photobiol 70 64–71 10420844

[R81] Hennig G, Stepp H and Johansson A 2011 Photobleaching-based method to individualize irradiation time during interstitial 5-aminolevulinic acid photodynamic therapy Photodiagn. Photodyn. Ther 8 275–81 10.1016/j.pdpdt.2011.03.338 21864802

[R82] Hryhorenko EA, Rittenhouse-Diakun K, Harvey NS, Morgan J, Stewart CC and Oseroff AR 1998 Characterization of endogenous protoporphyrin IX induced by delta-aminolevulinic acid in resting and activated peripheral blood lymphocytes by four-color flow cytometry Photochem. Photobiol 67 565–72 9613240

[R83] Huggett M, Jermyn M, Gillams A, Illing R, Mosse S, Novelli M, Kent E, Bown S, Hasan T, Pogue B and Pereira S 2014 Phase I/II study of verteporfin photodynamic therapy in locally advanced pancreatic cancer Br. J. Cancer at press 10.1038/bjc.2014.95 PMC3974098 24569464

[R84] Ickowicz Schwartz D, Gozlan Y, Greenbaum L, Babushkina T, Katcoff DJ and Malik Z 2004 Differentiation-dependent photodynamic therapy regulated by porphobilinogen deaminase in B16 melanoma Br. J. Cancer 90 1833–41 15150593 10.1038/sj.bjc.6601760 PMC2409749

[R85] Iinuma S, Schomacker KT, Wagnieres G, Rajadhyaksha M, Bamberg M, Momma T and Hasan T 1999 *In vivo* fluence rate and fractionation effects on tumor response and photobleaching: photodynamic therapy with two photosensitizers in an orthotopic rat tumor model Cancer Res. 59 6164–70 10626808

[R86] Jarvi MT, Niedre MJ, Patterson MS and Wilson BC 2006 Singlet oxygen luminescence dosimetry (SOLD) for photodynamic therapy: current status, challenges and future prospects Photochem. Photobiol 82 1198–210 16808593 10.1562/2006-05-03-IR-891

[R87] Jarvi MT, Niedre MJ, Patterson MS and Wilson BC 2011 The influence of oxygen depletion and photosensitizer triplet-state dynamics during photodynamic therapy on accurate singlet oxygen luminescence monitoring and analysis of treatment dose response Photochem. Photobiol 87 223–34 21143603 10.1111/j.1751-1097.2010.00851.x

[R88] Jarvi MT, Patterson MS and Wilson BC 2012 Insights into photodynamic therapy dosimetry: simultaneous singlet oxygen luminescence and photosensitizer photobleaching measurements Biophys. J 102 661–71 22325290 10.1016/j.bpj.2011.12.043 PMC3274798

[R89] Jermyn M, Davis SC, Dehghani H, Hugget M, Pereira SP, Bown S and Pogue BW 2014 CT contrast predicts pancreatic cancer treatment response to verteporfin-based photodynamic therapy Phys. Med. Biol 59 1911–21 24651456 10.1088/0031-9155/59/8/1911 PMC4229843

[R90] Ji Z, Yang G, Vasovic V, Cunderlikova B, Suo Z, Nesland JM and Peng Q 2006 Subcellular localization pattern of protoporphyrin IX is an important determinant for its photodynamic efficiency of human carcinoma and normal cell lines J. Photochem. Photobiol. B 84 213–20 16709459 10.1016/j.jphotobiol.2006.03.006

[R91] Jocham D, Baumgartner R, Stepp H and Unsold E 1990 Clinical experience with the integral photodynamic therapy of bladder carcinoma J. Photochem. Photobiol. B 6 183–7 2146378 10.1016/1011-1344(90)85088-e

[R92] Johansson A, Axelsson J, Andersson-Engels S and Swartling J 2007 Realtime light dosimetry software tools for interstitial photodynamic therapy of the human prostate Med. Phys 34 4309–21 18072496 10.1118/1.2790585

[R93] Johansson A, Faber F, Kniebuhler G, Stepp H, Sroka R, Egensperger R, Beyer W and Kreth FW 2013 Protoporphyrin IX fluorescence and photobleaching during interstitial photodynamic therapy of malignant gliomas for early treatment prognosis Lasers Surg. Med 45 225–34 23533060 10.1002/lsm.22126

[R94] Johansson T, Thompson MS, Stenberg M, af KC, Andersson-Engels S, Svanberg S and Svanberg K 2002 Feasibility study of a system for combined light dosimetry and interstitial photodynamic treatment of massive tumors Appl. Opt 41 1462–8 11900027 10.1364/ao.41.001462

[R95] Kabingu E, Oseroff AR, Wilding GE and Gollnick SO 2009 Enhanced systemic immune reactivity to a Basal cell carcinoma associated antigen following photodynamic therapy Clin. Cancer. Res 15 4460–6 19549769 10.1158/1078-0432.CCR-09-0400 PMC2805024

[R96] Kammerer R, Buchner A, Palluch P, Pongratz T, Oboukhovskij K, Beyer W, Johansson A, Stepp H, Baumgartner R and Zimmermann W 2011 Induction of immune mediators in glioma and prostate cancer cells by non-lethal photodynamic therapy PLoS One 6 e21834 21738796 10.1371/journal.pone.0021834 PMC3128096

[R97] Kanick SC, Davis SC, Zhao Y, Hasan T, Maytin EV, Pogue BW and Chapman MS 2014 Dual-channel red/blue fluorescence dosimetry with broadband reflectance spectroscopic correction measures protoporphyrin IX production during photodynamic therapy of actinic keratosis J. Biomed. Opt 19 75002 24996661 10.1117/1.JBO.19.7.075002 PMC4082494

[R98] Kanick SC, Davis SC, Zhao Y, Sheehan KL, Hasan T, Maytin EV, Pogue BW and Chapman MS 2015 Pre-treatment protoporphyrin IX concentration in actinic keratosis lesions may be a predictive biomarker of response to aminolevulinic-acid based photodynamic therapy Photodiagn. Photodyn. Ther 12 561–6 10.1016/j.pdpdt.2015.10.006 PMC4684466 26480810

[R99] Karakullukcu B 2013 MR and CT based treatment planning for mTHPC mediated interstitial photodynamic therapy of head and neck cancer: description of the method Lasers Surg. Med 45 517–23 24037957 10.1002/lsm.22174

[R100] Kelleher DK, Thews O, Scherz A, Salomon Y and Vaupel P 2004 Perfusion, oxygenation status and growth of experimental tumors upon photodynamic therapy with Pd-bacteriopheophorbide Int. J. Oncol 24 1505–11 15138594

[R101] Kennedy JC and Pottier RH 1992 Endogenous protoporphyrin IX, a clinically useful photosensitizer for photodynamic therapy J. Photochem. Photobiol. B 14 275–92 1403373 10.1016/1011-1344(92)85108-7

[R102] Kessel D 1992 Photodynamic therapy and neoplastic disease Oncol. Res 4 219–25 1421614

[R103] Kessel D and Poretz RD 2000 Sites of photodamage induced by photodynamic therapy with a chlorin e6 triacetoxymethyl ester (CAME) Photochem. Photobiol 71 94–6 10649895 10.1562/0031-8655(2000)071<0094:sopibp>2.0.co;2

[R104] Kety SS 1951 The theory and applications of the exchange of inert gas at the lungs and tissues Pharmacol. Rev 3 1–41 14833874

[R105] Korbelik M and Hamblin MR 2015 The impact of macrophage-cancer cell interaction on the efficacy of photodynamic therapy Photochem. Photobiol. Sci 14 1403–9 25620672 10.1039/c4pp00451e PMC4515410

[R106] Krieg RC, Messmann H, Schlottmann K, Endlicher E, Seeger S, Scholmerich J and Knuechel R 2003 Intracellular localization is a cofactor for the phototoxicity of protoporphyrin IX in the gastrointestinal tract: *in vitro* study Photochem. Photobiol 78 393–9 14626668 10.1562/0031-8655(2003)078<0393:iliacf>2.0.co;2

[R107] Kruijt B, van der Ploeg-van den Heuvel A, de Bruijn HS, Sterenborg HJ, Amelink A and Robinson DJ 2009 Monitoring interstitial m-THPC-PDT *in vivo* using fluorescence and reflectance spectroscopy Lasers Surg. Med 41 653–64 19802884 10.1002/lsm.20845

[R108] Kubler AC, Haase T, Staff C, Kahle B, Rheinwald M and Muhling J 1999 Photodynamic therapy of primary nonmelanomatous skin tumours of the head and neck Lasers Surg. Med 25 60–8 10421887 10.1002/(sici)1096-9101(1999)25:1<60::aid-lsm8>3.0.co;2-x

[R109] Kunz L, Connelly JP, Woodhams JH and MacRobert AJ 2007 Photodynamic modification of disulfonated aluminium phthalocyanine fluorescence in a macrophage cell line Photochem. Photobiol. Sci 6 940–8 17721592 10.1039/b708456k

[R110] Kunz L and MacRobert AJ 2002 Intracellular photobleaching of 5,10,15,20-tetrakis(m-hydroxyphenyl) chlorin (Foscan) exhibits a complex dependence on oxygen level and fluence rate Photochem. Photobiol 75 28–35 11837325 10.1562/0031-8655(2002)075<0028:ipotmh>2.0.co;2

[R111] Laubach HJ, Chang SK, Lee S, Rizvi I, Zurakowski D, Davis SJ, Taylor CR and Hasan T 2008 *In vivo* singlet oxygen dosimetry of clinical 5-aminolevulinic acid photodynamic therapy J. Biomed. Opt 13 050504 19021376 10.1117/1.2981813 PMC2994192

[R112] Lebedev AY, Cheprakov AV, Sakadzic S, Boas DA, Wilson DF and Vinogradov SA 2009 Dendritic phosphorescent probes for oxygen imaging in biological systems ACS Appl. Mater Interfaces 1 1292–304 20072726 10.1021/am9001698 PMC2805241

[R113] Lee S, Isabelle ME, Gabally-Kinney KL, Pogue BW and Davis SJ 2011 Dual-channel imaging system for singlet oxygen and photosensitizer for PDT Biomed. Opt. Express 2 1233–42 21559134 10.1364/BOE.2.001233 PMC3087579

[R114] Lerche CM, Fabricius S, Philipsen PA and Wulf HC 2015 Correlation between treatment time, photobleaching, inflammation and pain after photodynamic therapy with methyl aminolevulinate on tape-stripped skin in healthy volunteers Photochem. Photobiol. Sci 14 875–82 25812618 10.1039/c5pp00069f

[R115] Li J, Altschuler MD, Hahn SM and Zhu TC 2008 Optimization of light source parameters in the photodynamic therapy of heterogeneous prostate Phys. Med. Biol 53 4107–21 18612172 10.1088/0031-9155/53/15/007 PMC3276881

[R116] Li J and Zhu TC 2008 Determination of *in vivo* light fluence distribution in a heterogeneous prostate during photodynamic therapy Phys. Med. Biol 53 2103–14 18369279 10.1088/0031-9155/53/8/007 PMC3276882

[R117] Lilge L, Molpus K, Hasan T and Wilson BC 1998 Light dosimetry for intraperitoneal photodynamic therapy in a murine xenograft model of human epithelial ovarian carcinoma Photochem. Photobiol 68 281–8 9747583

[R118] Lilge L and Wilson BC 1998 Photodynamic therapy of intracranial tissues: a preclinical comparative study of four different photosensitizers J. Clin. Laser Med. Surg 16 81–91 9663099 10.1089/clm.1998.16.81

[R119] Lin JC and Song CW 1993 Influence of vascular thermotolerance on the heat-induced changes in blood flow, pO_2_, and cell survival in tumors Cancer Res. 53 2076–80 8481910

[R120] Mackenzie GD, Jamieson NF, Novelli MR, Mosse CA, Clark BR, Thorpe SM, Bown SG and Lovat LB 2008 How light dosimetry influences the efficacy of photodynamic therapy with 5-aminolaevulinic acid for ablation of high-grade dysplasia in Barrett’s esophagus Lasers Med. Sci 23 203–10 17610005 10.1007/s10103-007-0473-7

[R121] Maeda H, Sawa T and Konno T 2001 Mechanism of tumor-targeted delivery of macromolecular drugs, including the EPR effect in solid tumor and clinical overview of the prototype polymeric drug SMANCS J. Control Release 74 47–61 11489482 10.1016/s0168-3659(01)00309-1

[R122] Mallidi S, Anbil S, Lee S, Manstein D, Elrington S, Kositratna G, Schoenfeld D, Pogue B, Davis SJ and Hasan T 2014 Photosensitizer fluorescence and singlet oxygen luminescence as dosimetric predictors of topical 5-aminolevulinic acid photodynamic therapy induced clinical erythema J. Biomed. Opt 19 028001 24503639 10.1117/1.JBO.19.2.028001 PMC3915169

[R123] Marijnissen JP, Baas P, Beek JF, van Moll JH, van Zandwijk N and Star WM 1993 Pilot study on light dosimetry for endobronchial photodynamic therapy Photochem. Photobiol 58 92–9 8378437 10.1111/j.1751-1097.1993.tb04908.x

[R124] Marynissen JP, Jansen H and Star WM 1989 Treatment system for whole bladder wall photodynamic therapy with *in vivo* monitoring and control of light dose rate and dose J. Urol 142 1351–5 2810531 10.1016/s0022-5347(17)39096-1

[R125] McIlroy BW, Curnow A, Buonaccorsi G, Scott MA, Bown SG and MacRobert AJ 1998 Spatial measurement of oxygen levels during photodynamic therapy using time-resolved optical spectroscopy J. Photochem. Photobiol. B 43 47–55 9639914 10.1016/s1011-1344(98)00081-5

[R126] McLoone N, Donnelly RF, Walsh M, Dolan OM, McLoone S, McKenna K and McCarron PA 2008 Aminolaevulinic acid diffusion characteristics in ‘*in vitro*’ normal human skin and actinic keratosis: implications for topical photodynamic therapy Photodermatol. Photoimmunol. Photomed 24 183–90 18717959 10.1111/j.1600-0781.2008.00358.x

[R127] Mitra S, Modi KD and Foster TH 2013 Enzyme-activatable imaging probe reveals enhanced neutrophil elastase activity in tumors following photodynamic therapy J. Biomed. Opt 18 101314 23897439 10.1117/1.JBO.18.10.101314 PMC3726228

[R128] Niedre M, Patterson MS and Wilson BC 2002 Direct near-infrared luminescence detection of singlet oxygen generated by photodynamic therapy in cells *in vitro* and tissues *in vivo* Photochem. Photobiol 75 382–91 12003128 10.1562/0031-8655(2002)075<0382:DNILDO>2.0.CO;2

[R129] Niedre MJ, Yu CS, Patterson MS and Wilson BC 2005 Singlet oxygen luminescence as an *in vivo* photodynamic therapy dose metric: validation in normal mouse skin with topical amino-levulinic acid Br. J. Cancer 92 298–304 15655542 10.1038/sj.bjc.6602331 PMC2361839

[R130] Ochsner M 1997 Photophysical and photobiological processes in the photodynamic therapy of tumours J. Photochem. Photobiol. B 39 1–18 9210318 10.1016/s1011-1344(96)07428-3

[R131] Okihara K, Watanabe H and Kojima M 1999 Kinetic study of tumor blood flow in prostatic cancer using power Doppler imaging Ultrasound Med. Biol 25 89–94 10048805 10.1016/s0301-5629(98)00140-9

[R132] Ortel B, Sharlin D, O’Donnell D, Sinha AK, Maytin EV and Hasan T 2002 Differentiation enhances aminolevulinic acid-dependent photodynamic treatment of LNCaP prostate cancer cells Br. J. Cancer 87 1321–7 12439724 10.1038/sj.bjc.6600575 PMC2408893

[R133] Osaki T, Takagi S, Hoshino Y, Okumura M and Fujinaga T 2006 Intracellular localization and concentration as well as photodynamic effects of benzoporphyrin derivative monoacid ring a in four types of rodent tumor cells Cancer Lett. 243 281–92 16412570 10.1016/j.canlet.2005.11.044

[R134] Panjehpour M, Overholt BF, Phan MN and Haydek JM 2005 Optimization of light dosimetry for photodynamic therapy of Barrett’s esophagus: efficacy versus incidence of stricture after treatment Gastrointest. Endosc 61 13–8 15672050 10.1016/s0016-5107(04)02394-6

[R135] Patel G, Armstrong AW and Eisen DB 2014 Efficacy of photodynamic therapy versus other interventions in randomized clinical trials for the treatment of actinic keratoses: a systematic review and meta-analysis JAMA Dermatol. 150 1281–8 25162181 10.1001/jamadermatol.2014.1253

[R136] Patlak CS, Blasberg RG and Fenstermacher JD 1983 Graphical evaluation of blood-to-brain transfer constants from multiple-time uptake data J. Cereb. Blood Flow Metab 3 1–7 6822610 10.1038/jcbfm.1983.1

[R137] Patterson MS, Madsen SJ and Wilson BC 1990a Experimental tests of the feasibility of singlet oxygen luminescence monitoring *in vivo* during photodynamic therapy J. Photochem. Photobiol. B 5 69–84 2111394 10.1016/1011-1344(90)85006-i

[R138] Patterson MS, Wilson BC and Graff R 1990b *In vivo* tests of the concept of photodynamic threshold dose in normal rat liver photosensitized by aluminum chlorosulphonated phthalocyanine Photochem. Photobiol 51 343–9 2356229 10.1111/j.1751-1097.1990.tb01720.x

[R139] Peters-Engl C, Frank W, Leodolter S and Medl M 1999 Tumor flow in malignant breast tumors measured by Doppler ultrasound: an independent predictor of survival Breast Cancer Res. Treat 54 65–71 10369082 10.1023/a:1006148812831

[R140] Pham TH, Hornung R, Berns MW, Tadir Y and Tromberg BJ 2001 Monitoring tumor response during photodynamic therapy using near-infrared photon-migration spectroscopy Photochem. Photobiol 73 669–77 11421074 10.1562/0031-8655(2001)073<0669:mtrdpt>2.0.co;2

[R141] Pogue BW, Braun RD, Lanzen JL, Erickson C and Dewhirst MW 2001a Analysis of the heterogeneity of pO_2_ dynamics during photodynamic therapy with verteporfin Photochem. Photobiol 74 700–6 11723798 10.1562/0031-8655(2001)074<0700:aothop>2.0.co;2

[R142] Pogue BW, Ortel B, Chen N, Redmond RW and Hasan T 2001b A photobiological and photophysical-based study of phototoxicity of two chlorins Cancer Res. 61 717–24 11212274

[R143] Pogue BW, Sheng C, Benevides J, Forcione D, Puricelli B, Nishioka N and Hasan T 2008 Protoporphyrin IX fluorescence photobleaching increases with the use of fractionated irradiation in the esophagus J. Biomed. Opt 13 034009 18601554 10.1117/1.2937476 PMC3787899

[R144] Potter WR, Mang TS and Dougherty TJ 1987 The theory of photodynamic therapy dosimetry: consequences of photo-destruction of sensitizer Photochem. Photobiol 46 97–101 2956621 10.1111/j.1751-1097.1987.tb04741.x

[R145] Powers SK and Brown JT 1986 Light dosimetry in brain tissue: an *in vivo* model applicable to photodynamic therapy Lasers Surg. Med 6 318–22 2942742 10.1002/lsm.1900060305

[R146] Radu A, Conde R, Fontolliet C, Wagnieres G, Van den Bergh H and Monnier P 2003 Mucosal ablation with photodynamic therapy in the esophagus: optimization of light dosimetry in the sheep model Gastrointest. Endosc 57 897–905 12776039 10.1016/s0016-5107(03)70027-3

[R147] Redmond RW, Srichai MB, Bilitz JM, Schlomer DD and Krieg M 1994 Merocyanine dyes: effect of structural modifications on photophysical properties and biological activity Photochem. Photobiol 60 348–55 7527561 10.1111/j.1751-1097.1994.tb05114.x

[R148] Rizvi I, Anbil S, Alagic N, Celli J, Zheng LZ, Palanisami A, Glidden MD, Pogue BW and Hasan T 2013 PDT dose parameters impact tumoricidal durability and cell death pathways in a 3D ovarian cancer model Photochem. Photobiol 89 942–52 23442192 10.1111/php.12065 PMC3701746

[R149] Robinson DJ, de Bruijn HS, de Wolf WJ, Sterenborg HJ and Star WM 2000 Topical 5-aminolevulinic acid-photodynamic therapy of hairless mouse skin using two-fold illumination schemes: PpIX fluorescence kinetics, photobleaching and biological effect Photochem. Photobiol 72 794–802 11140268 10.1562/0031-8655(2000)072<0794:TAAPTO>2.0.CO;2

[R150] Robinson DJ, de Bruijn HS, van der Veen N, Stringer MR, Brown SB and Star WM 1998 Fluorescence photobleaching of ALA-induced protoporphyrin IX during photodynamic therapy of normal hairless mouse skin: the effect of light dose and irradiance and the resulting biological effect Photochem. Photobiol 67 140–9 9477772

[R151] Robinson DJ, de Bruijn HS, van der Veen N, Stringer MR, Brown SB and Star WM 1999 Protoporphyrin IX fluorescence photobleaching during ALA-mediated photodynamic therapy of UVB-induced tumors in hairless mouse skin Photochem. Photobiol 69 61–70 10063801

[R152] Rollakanti KR, Kanick SC, Davis SC, Pogue BW and Maytin EV 2013 Techniques for fluorescence detection of protoporphyrin IX in skin cancers associated with photodynamic therapy Photonics Lasers Med. 2 287–303 25599015 10.1515/plm-2013-0030 PMC4295789

[R153] Rosen MA and Schnall MD 2007 Dynamic contrast-enhanced magnetic resonance imaging for assessing tumor vascularity and vascular effects of targeted therapies in renal cell carcinoma Clin. Cancer Res 13 770s–6s 17255308 10.1158/1078-0432.CCR-06-1921

[R154] Sahani DV, Kalva SP, Hamberg LM, Hahn PF, Willett CG, Saini S, Mueller PR and Lee TY 2005 Assessing tumor perfusion and treatment response in rectal cancer with multisection CT: initial observations Radiology 234 785–92 15734934 10.1148/radiol.2343040286

[R155] Sailer R, Strauss WS, Wagner M, Emmert H and Schneckenburger H 2007 Relation between intracellular location and photodynamic efficacy of 5-aminolevulinic acid-induced protoporphyrin IX *in vitro*. Comparison between human glioblastoma cells and other cancer cell lines Photochem. Photobiol. Sci 6 145–51 17277837 10.1039/b611715e

[R156] Sasnouski S, Kachatkou D, Zorin V, Guillemin F and Bezdetnaya L 2006 Redistribution of Foscan from plasma proteins to model membranes Photochem. Photobiol. Sci 5 770–7 16886093 10.1039/b603840a

[R157] Schick E, Kaufmann R, Ruck A, Hainzl A and Boehncke WH 1995 Influence of activation and differentiation of cells on the effectiveness of photodynamic therapy Acta Derm. Venereol 75 276–9 8578947 10.2340/0001555575276279

[R158] Schmidt-Erfurth U, Hasan T, Schomacker K, Flotte T and Birngruber R 1995 *In vivo* uptake of liposomal benzoporphyrin derivative and photothrombosis in experimental corneal neovascularization Lasers Surg. Med 17 178–88 8569414 10.1002/lsm.1900170207

[R159] Seidl J, Rauch J, Krieg RC, Appel S, Baumgartner R and Knuechel R 2001 Optimization of differential photodynamic effectiveness between normal and tumor urothelial cells using 5-aminolevulinic acid-induced protoporphyrin IX as sensitizer Int. J. Cancer 92 671–7 11340570 10.1002/1097-0215(20010601)92:5<671::aid-ijc1240>3.0.co;2-p

[R160] Shams M, Owczarczak B, Manderscheid-Kern P, Bellnier DA and Gollnick SO 2015 Development of photodynamic therapy regimens that control primary tumor growth and inhibit secondary disease Cancer Immunol. Immunother 64 287–97 25384911 10.1007/s00262-014-1633-9 PMC4341021

[R161] Sharwani A and Alharbi FA 2014 Monitoring of photobleaching in photodynamic therapy using fluorescence spectroscopy Gulf J. Oncol 1 79–83 25316396

[R162] Sheng C 2006 Dosimetry for 5-Aminolevulinic acid induced protoporphyrin IX photodynamic therapy of Barrett’s Esophagus Thayer School of Engineering (Hanover, NH: Dartmouth College)

[R163] Sheng C, Hoopes PJ, Hasan T and Pogue BW 2007 Photobleaching-based dosimetry predicts deposited dose in ALA-PpIX PDT of rodent esophagus Photochem. Photobiol 83 738–48 17576383 10.1562/2006-09-07-RA-1033

[R164] Silva AC, Kim SG and Garwood M 2000 Imaging blood flow in brain tumors using arterial spin labeling Magn. Reson. Med 44 169–73 10918313 10.1002/1522-2594(200008)44:2<169::aid-mrm1>3.0.co;2-u

[R165] Sitnik TM, Hampton JA and Henderson BW 1998 Reduction of tumour oxygenation during and after photodynamic therapy *in vivo*: effects of fluence rate Br. J. Cancer 77 1386–94 9652753 10.1038/bjc.1998.231 PMC2150183

[R166] Solonenko M, Cheung R, Busch TM, Kachur A, Griffin GM, Vulcan T, Zhu TC, Wang HW, Hahn SM and Yodh AG 2002 *In vivo* reflectance measurement of optical properties, blood oxygenation and motexafin lutetium uptake in canine large bowels, kidneys and prostates Phys. Med. Biol 47 857–73 11936174

[R167] Song CW, Shakil A, Osborn JL and Iwata K 1996 Tumour oxygenation is increased by hyperthermia at mild temperatures Int. J. Hyperth 12 367–73 10.3109/02656739609022525 9044906

[R168] Sourbron SP and Buckley DL 2011 Tracer kinetic modelling in MRI: estimating perfusion and capillary permeability Phys. Med. Biol 57 R1–33 22173205 10.1088/0031-9155/57/2/R1

[R169] Star WM, Marijnissen HP, Jansen H, Keijzer M and van Gemert MJ 1987 Light dosimetry for photodynamic therapy by whole bladder wall irradiation Photochem. Photobiol 46 619–24 3441490 10.1111/j.1751-1097.1987.tb04822.x

[R170] Strauss WS, Gschwend MH, Sailer R, Schneckenburger H, Steiner R and Ruck A 1995 Intracellular fluorescence behaviour of meso-tetra(4-sulphonatophenyl)porphyrin during photodynamic treatment at various growth phases of cultured cells J. Photochem. Photobiol. B 28 155–61 7636637 10.1016/1011-1344(94)07082-y

[R171] Sunar U 2013 Monitoring photodynamic therapy of head and neck malignancies with optical spectroscopies World J. Clin. Cases 1 96–105 24303476 10.12998/wjcc.v1.i3.96 PMC3845916

[R172] Svaasand LO, Wyss P, Wyss MT, Tadir Y, Tromberg BJ and Berns MW 1996 Dosimetry model for photodynamic therapy with topically administered photosensitizers Lasers Surg. Med 18 139–49 8833282 10.1002/(SICI)1096-9101(1996)18:2<139::AID-LSM3>3.0.CO;2-T

[R173] Svanberg K 1998 Clinical multi-colour fluorescence imaging of malignant tumours--initial experience Acta Radiol. 39 2–9 9498864 10.1080/02841859809172141

[R174] Swartling J, Axelsson J, Ahlgren G, Kalkner KM, Nilsson S, Svanberg S, Svanberg K and Andersson-Engels S 2010 System for interstitial photodynamic therapy with online dosimetry: first clinical experiences of prostate cancer J. Biomed. Opt 15 058003 21054129 10.1117/1.3495720

[R175] Tan IB, Oppelaar H, Ruevekamp MC, Veenhuizen RB, Timmers A and Stewart FA 1999 The importance of *in situ* light dosimetry for photodynamic therapy of oral cavity tumors Head Neck 21 434–41 10402524 10.1002/(sici)1097-0347(199908)21:5<434::aid-hed9>3.0.co;2-b

[R176] Thews O, Kelleher DK and Vaupel P 2000 Disparate responses of tumour vessels to angiotensin II: tumour volume-dependent effects on perfusion and oxygenation Br. J. Cancer 83 225–31 10901375 10.1054/bjoc.2000.1229 PMC2363484

[R177] Thews O, Kelleher DK and Vaupel P 2001 No improvement in perfusion and oxygenation of experimental tumors upon application of vasodilator drugs Int. J. Oncol 19 1243–7 11713595 10.3892/ijo.19.6.1243

[R178] Thompson MS, Johansson A, Johansson T, Andersson-Engels S, Svanberg S, Bendsoe N and Svanberg K 2005 Clinical system for interstitial photodynamic therapy with combined on-line dosimetry measurements Appl. Opt 44 4023–31 16004049 10.1364/ao.44.004023

[R179] Trachtenberg J 2007 Vascular targeted photodynamic therapy with palladium-bacteriopheophorbide photosensitizer for recurrent prostate cancer following definitive radiation therapy: assessment of safety and treatment response J. Urol 178 1974–9 discussion 9 17869307 10.1016/j.juro.2007.07.036

[R180] Tromberg BJ, Orenstein A, Kimel S, Barker SJ, Hyatt J, Nelson JS and Berns MW 1990 *In vivo* tumor oxygen tension measurements for the evaluation of the efficiency of photodynamic therapy Photochem. Photobiol 52 375–85 2145595 10.1111/j.1751-1097.1990.tb04193.x

[R181] Tromberg BJ, Svaasand LO, Fehr MK, Madsen SJ, Wyss P, Sansone B and Tadir Y 1996 A mathematical model for light dosimetry in photodynamic destruction of human endometrium Phys. Med. Biol 41 223–37 8746106 10.1088/0031-9155/41/2/002

[R182] Tyrrell J, Campbell S and Curnow A 2010a Protoporphyrin IX photobleaching during the light irradiation phase of standard dermatological methyl-aminolevulinate photodynamic therapy Photodiagn. Photodyn. Ther 7 232–8 10.1016/j.pdpdt.2010.09.005 21112545

[R183] Tyrrell JS, Campbell SM and Curnow A 2010b The relationship between protoporphyrin IX photobleaching during real-time dermatological methyl-aminolevulinate photodynamic therapy (MAL-PDT) and subsequent clinical outcome Lasers Surg. Med 42 613–9 20806386 10.1002/lsm.20943

[R184] Valentine RM, Ibbotson SH, Brown CT, Wood K and Moseley H 2011 A quantitative comparison of 5-aminolaevulinic acid- and methyl aminolevulinate-induced fluorescence, photobleaching and pain during photodynamic therapy Photochem. Photobiol 87 242–9 21077899 10.1111/j.1751-1097.2010.00829.x

[R185] Valentine RM, Ibbotson SH, Wood K, Brown CT and Moseley H 2013 Modelling fluorescence in clinical photodynamic therapy Photochem. Photobiol. Sci 12 203–13 23128146 10.1039/c2pp25271f

[R186] Van der Veen N, De Bruijn HS and Star WM 1997 Photobleaching during and re-appearance after photodynamic therapy of topical ALA-induced fluorescence in UVB-treated mouse skin Int. J. Cancer 72 110–8 9212231 10.1002/(sici)1097-0215(19970703)72:1<110::aid-ijc16>3.0.co;2-n

[R187] van Duijnhoven FH, Aalbers RI, Rovers JP, Terpstra OT and Kuppen PJ 2003 The immunological consequences of photodynamic treatment of cancer, a literature review Immunobiology 207 105–13 12675268 10.1078/0171-2985-00221

[R188] van Veen RL, Aalders MC, Pasma KL, Siersema PD, Haringsma J, van de Vrie W, Gabeler EE, Robinson DJ and Sterenborg HJ 2002 *In situ* light dosimetry during photodynamic therapy of Barrett’s esophagus with 5-aminolevulinic acid Lasers Surg. Med 31 299–304 12430146 10.1002/lsm.10129

[R189] van Veen RL, Robinson DJ, Siersema PD and Sterenborg HJ 2006 The importance of *in situ* dosimetry during photodynamic therapy of Barrett’s esophagus Gastrointest. Endosc 64 786–8 17055875 10.1016/j.gie.2006.06.056

[R190] Vaupel P, Hockel M and Mayer A 2007 Detection and characterization of tumor hypoxia using pO2 histography Antioxid Redox Signal 9 1221–35 17536958 10.1089/ars.2007.1628

[R191] Vaupel P, Mayer A, Briest S and Hockel M 2005 Hypoxia in breast cancer: role of blood flow, oxygen diffusion distances, and anemia in the development of oxygen depletion Adv. Exp. Med. Biol 566 333–42 16594170 10.1007/0-387-26206-7_44

[R192] Vaupel P, Mayer A and Hockel M 2006 Oxygenation status of primary and recurrent squamous cell carcinomas of the vulva Eur. J. Gynaecol. Oncol 27 142–6 16620056

[R193] Vaupel PW and Kelleher DK 2010 Pathophysiological and vascular characteristics of tumours and their importance for hyperthermia: heterogeneity is the key issue Int. J. Hyperth 26 211–23 10.3109/02656731003596259 20345270

[R194] Vujaskovic Z 2000 Temperature-dependent changes in physiologic parameters of spontaneous canine soft tissue sarcomas after combined radiotherapy and hyperthermia treatment Int. J. Radiat. Oncol. Biol. Phys 46 179–85 10656391 10.1016/s0360-3016(99)00362-4

[R195] Wallace MJ, Kuo MD, Glaiberman C, Binkert CA, Orth RC and Soulez G 2008 3D C-arm cone-beam CT: applications in the interventional suite J. Vasc. Interv. Radiol 19 799–813 18503893 10.1016/j.jvir.2008.02.018

[R196] Wang HW 2007 Quantitative comparison of tissue oxygen and motexafin lutetium uptake by *ex vivo* and noninvasive *in vivo* techniques in patients with intraperitoneal carcinomatosis J. Biomed. Opt 12 034023 17614731 10.1117/1.2743082

[R197] Wang HW, Putt ME, Emanuele MJ, Shin DB, Glatstein E, Yodh AG and Busch TM 2004 Treatment-induced changes in tumor oxygenation predict photodynamic therapy outcome Cancer Res. 64 7553–61 15492282 10.1158/0008-5472.CAN-03-3632

[R198] Wang HW 2005 Broadband reflectance measurements of light penetration, blood oxygenation, hemoglobin concentration, and drug concentration in human intraperitoneal tissues before and after photodynamic therapy J. Biomed. Opt 10 14004 15847585 10.1117/1.1854679

[R199] Wang KK, Cottrell WJ, Mitra S, Oseroff AR and Foster TH 2009 Simulations of measured photobleaching kinetics in human basal cell carcinomas suggest blood flow reductions during ALA-PDT Lasers Surg. Med 41 686–96 19802891 10.1002/lsm.20847 PMC2805271

[R200] Wang KK, Finlay JC, Busch TM, Hahn SM and Zhu TC 2010 Explicit dosimetry for photodynamic therapy: macroscopic singlet oxygen modeling J. Biophotonics 3 304–18 20222102 10.1002/jbio.200900101 PMC3071971

[R201] Wang KK and Zhu TC 2009 Reconstruction of *in vivo* optical properties for human prostate using interstitial diffuse optical tomography Opt. Express 17 11665–72 19582081 10.1364/oe.17.011665 PMC3276880

[R202] Warren CB, Lohser S, Wene LC, Pogue BW, Bailin PL and Maytin EV 2010 Noninvasive fluorescence monitoring of protoporphyrin IX production and clinical outcomes in actinic keratoses following short-contact application of 5-aminolevulinate J. Biomed. Opt 15 051607 21054081 10.1117/1.3484255 PMC2955723

[R203] Weersink RA, Bogaards A, Gertner M, Davidson SR, Zhang K, Netchev G, Trachtenberg J and Wilson BC 2005 Techniques for delivery and monitoring of TOOKAD (WST09)-mediated photodynamic therapy of the prostate: clinical experience and practicalities J. Photochem. Photobiol. B 79 211–22 15896648 10.1016/j.jphotobiol.2005.01.008

[R204] Wei Y, Xing D, Luo S, Xu W and Chen Q 2008 Monitoring singlet oxygen *in situ* with delayed chemiluminescence to deduce the effect of photodynamic therapy J. Biomed. Opt 13 024023 18465986 10.1117/1.2904961

[R205] Wenig BL, Kurtzman DM, Grossweiner LI, Mafee MF, Harris DM, Lobraico RV, Prycz RA and Appelbaum EL 1990 Photodynamic therapy in the treatment of squamous cell carcinoma of the head and neck Arch. Otolaryngol. Head Neck Surg 116 1267–70 2146969 10.1001/archotol.1990.01870110039003

[R206] Werkhaven J, Harris DM, Krol G and Hill JH 1986 Light dosimetry in animal models: application to photodynamic therapy in otolaryngology Laryngoscope 96 1058–61 3762280 10.1288/00005537-198610000-00002

[R207] Weston MA and Patterson MS 2013 Monitoring oxygen concentration during photodynamic therapy using prompt photosensitizer fluorescence Phys. Med. Biol 58 7039–59 24051952 10.1088/0031-9155/58/20/7039

[R208] Wiegell SR 2012 Daylight-mediated photodynamic therapy of moderate to thick actinic keratoses of the face and scalp: a randomized multicentre study Br. J. Dermatol 166 1327–32 22250644 10.1111/j.1365-2133.2012.10833.x

[R209] Wiegell SR 2011 A randomized, multicentre study of directed daylight exposure times of 1(1/2) versus 2(1/2) h in daylight-mediated photodynamic therapy with methyl aminolaevulinate in patients with multiple thin actinic keratoses of the face and scalp Br. J. Dermatol 164 1083–90 21219287 10.1111/j.1365-2133.2011.10209.x

[R210] Wieman TJ and Fingar VH 1992 Photodynamic therapy Surg. Clin. North Am 72 609–22 1589835 10.1016/s0039-6109(16)45736-1

[R211] Wilson BC, Muller PJ and Yanch JC 1986 Instrumentation and light dosimetry for intra-operative photodynamic therapy (PDT) of malignant brain tumours Phys. Med. Biol 31 125–33 3008201 10.1088/0031-9155/31/2/002

[R212] Wilson BC and Patterson MS 1986 The physics of photodynamic therapy Phys. Med. Biol 31 327–60 3526361 10.1088/0031-9155/31/4/001

[R213] Wilson BC and Patterson MS 2008 The physics, biophysics and technology of photodynamic therapy Phys. Med. Biol 53 R61–109 18401068 10.1088/0031-9155/53/9/R01

[R214] Wilson BC, Patterson MS and Lilge L 1997 Implicit and explicit dosimetry in photodynamic therapy: a new paradigm Lasers Med. Sci 12 182–99 20803326 10.1007/BF02765099

[R215] Wilson DF, Harrison DK and Vinogradov SA 2012 Oxygen, pH, and mitochondrial oxidative phosphorylation J. Appl. Physiol 113 1838–45 23104697 10.1152/japplphysiol.01160.2012

[R216] Wilson EC 2010 Cost effectiveness of imiquimod 5% cream compared with methyl aminolevulinate-based photodynamic therapy in the treatment of non-hyperkeratotic, nonhypertrophic actinic (solar) keratoses: a decision tree model Pharmacoeconomics 28 1055–64 20936887 10.2165/11538670-000000000-00000

[R217] Wong TW, Tracy E, Oseroff AR and Baumann H 2003 Photodynamic therapy mediates immediate loss of cellular responsiveness to cytokines and growth factors Cancer Res. 63 3812–8 12839978

[R218] Woodhams J, Lou PJ, Selbo PK, Mosse A, Oukrif D, MacRobert A, Novelli M, Peng Q, Berg K and Bown SG 2010 Intracellular re-localisation by photochemical internalisation enhances the cytotoxic effect of gelonin—quantitative studies in normal rat liver J. Control Release 142 347–53 19932724 10.1016/j.jconrel.2009.11.017

[R219] Woodhams JH, Macrobert AJ and Bown SG 2007 The role of oxygen monitoring during photodynamic therapy and its potential for treatment dosimetry Photochem. Photobiol. Sci 6 1246–56 18046479 10.1039/b709644e

[R220] Xiang L, Xing D, Gu H, Yang D, Yang S, Zeng L and Chen WR 2007 Real-time optoacoustic monitoring of vascular damage during photodynamic therapy treatment of tumor J. Biomed. Opt 12 014001 17343476 10.1117/1.2437752

[R221] Yang VX, Muller PJ, Herman P and Wilson BC 2003 A multispectral fluorescence imaging system: design and initial clinical tests in intra-operative Photofrin-photodynamic therapy of brain tumors Lasers Surg. Med 32 224–32 12605430 10.1002/lsm.10131

[R222] Yu G, Durduran T, Zhou C, Zhu TC, Finlay JC, Busch TM, Malkowicz SB, Hahn SM and Yodh AG 2006 Real-time *in situ* monitoring of human prostate photodynamic therapy with diffuse light Photochem. Photobiol 82 1279–84 16696593 10.1562/2005-10-19-RA-721

[R223] Zeitouni NC, Sunar U, Rohrbach DJ, Paquette AD, Bellnier DA, Shi Y, Wilding G, Foster TH and Henderson BW 2014 A prospective study of pain control by a 2-step irradiance schedule during topical photodynamic therapy of nonmelanoma skin cancer Dermatol. Surg 40 1390–94 25393353 10.1097/DSS.0000000000000183 PMC4323090

[R224] Zeng H, Korbelik M, McLean DI, MacAulay C and Lui H 2002 Monitoring photoproduct formation and photobleaching by fluorescence spectroscopy has the potential to improve PDT dosimetry with a verteporfin-like photosensitizer Photochem. Photobiol 75 398–405 12003130 10.1562/0031-8655(2002)075<0398:mpfapb>2.0.co;2

[R225] Zhou X, Chen B, Hoopes PJ, Hasan T and Pogue BW 2006 Tumor vascular area correlates with photosensitizer uptake: analysis of verteporfin microvascular delivery in the dunning rat prostate tumor Photochem. Photobiol 82 1348–57 17421078 10.1562/2006-03-25-ra-858

[R226] Zhu TC 2012 Dosimetry in pleural photodynamic therapy J. Natl Compr. Cancer Netw 10 S60–4 10.6004/jnccn.2012.0178 23055219

[R227] Zhu TC and Finlay JC 2006 Prostate PDT dosimetry Photodiagn. Photodyn. Ther 3 234–46 10.1016/j.pdpdt.2006.08.002 PMC4469490 25046988

[R228] Zhu TC, Finlay JC and Hahn SM 2005 Determination of the distribution of light, optical properties, drug concentration, and tissue oxygenation *in vivo* in human prostate during motexafin lutetium-mediated photodynamic therapy J. Photochem. Photobiol. B 79 231–41 15896650 10.1016/j.jphotobiol.2004.09.013 PMC4470428

[R229] Zhu TC, Liang X, Kim MM, Finlay JC, Dimofte A, Rodriguez C, Simone CB, Friedberg JS 2nd and Cengel KA 2015 An IR navigation system for pleural PDT Front. Phys 3 9 25995987 10.3389/fphy.2015.00009 PMC4435962

[R230] Zilberstein J, Bromberg A, Frantz A, Rosenbach-Belkin V, Kritzmann A, Pfefermann R, Salomon Y and Scherz A 1997 Light-dependent oxygen consumption in bacteriochlorophyll-serine-treated melanoma tumors: on-line determination using a tissue-inserted oxygen microsensor Photochem. Photobiol 65 1012–9 9188281 10.1111/j.1751-1097.1997.tb07962.x

